# Multiple Key Hosts and Network Structure Shape Viral Prevalence Across Multispecies Communities of Bees

**DOI:** 10.1111/ele.70327

**Published:** 2026-01-28

**Authors:** Patrycja Pluta, Annika L. Hass, Kathrin Czechofsky, Catrin Westphal, Robert J. Paxton

**Affiliations:** ^1^ General Zoology, Institute for Biology Martin Luther University Halle‐Wittenberg Halle (Saale) Germany; ^2^ Functional Agrobiodiversity & Agroecology, Department of Crop Sciences University of Göttingen Göttingen Germany; ^3^ Centre of Biodiversity and Sustainable Land Use University of Göttingen Göttingen Germany; ^4^ German Centre for Integrative Biodiversity Research (iDiv) Halle‐Jena‐Leipzig Leipzig Germany

**Keywords:** basic reproduction number, bee, ecology, epidemiology, interaction network, key host, network structure, spillover, virus

## Abstract

Emerging infectious diseases (EIDs) threaten biodiversity, yet identifying key host species in complex ecological communities remains a major challenge. Here, we develop a quantitative framework combining field data, epidemiological modelling, simulations, and Bayesian inference to pinpoint key viral hosts in multispecies bee communities. Using flower–visitor interaction data and molecular virus screening, we estimate species‐specific basic reproduction numbers (*R*_0_) and assess the role of both key hosts and community metrics in virus transmission and persistence. We show that, while honeybees often act as primary reservoirs for deformed wing virus and black queen cell virus, others, such as the bumblebee *Bombus lapidarius* , can drive the spread of acute bee paralysis virus. Viral dynamics are primarily explained by exposure to key hosts, while community effects are not as pronounced. Identification of non‐honeybee key hosts challenges existing assumptions and highlights drivers of transmission and pathogen persistence in complex host–pathogen networks.

## Graphical abstract

This study develops a quantitative framework that integrates field data, epidemiological models, simulations, and Bayesian inference to identify key viral hosts in multispecies bee communities. By estimating species‐specific *R*_0_ values from flower‐visitor networks and viral screening, we show that honeybees, as well as other wild bees, can drive the spread of viruses. The findings demonstrate that exposure to key hosts primarily explains viral dynamics.

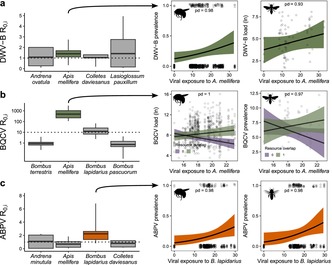

## 1 Introduction

Emerging infectious diseases (EIDs) exert a profound impact on wildlife, compromising individual health and eroding biodiversity (Daszak et al. 2000; Mahon et al. 2024). Disease emergence may subsequently lead to spillover, the transmission of a pathogen from a key host species (Box 1) to an alternative host species (Becker et al. 2019; Power and Mitchell 2004). Domestic animals often serve as important hosts for viral pathogens. For example, in the Serengeti, rabies and canine distemper viruses are spread from domestic dogs to wild carnivores (Viana et al. 2015). This shows that pathogens and their hosts usually exist within multispecies communities (Fenton et al. 2015; Rigaud et al. 2010). Identifying key hosts is therefore pivotal to understanding the epidemiology of pathogens and designing effective and efficient means to control them, yet it remains a major challenge (Streicker et al. 2013; Viana et al. 2014).

BOX 1 **Glossary.**

1

**Table:** 

Term	Definition
Key host	The host species that has the greatest potential for spreading a pathogen (highest *R*_0,i_ for the pathogen).
Viral prevalence	The proportion of individuals that are positive for a virus.
Basic reproduction number (*R*_0_)	Number of cases generated by one infected individual in an all‐susceptible population. *R*_0,i_ is the species‐specific basic reproduction number of species *i* that accounts for the contribution of other species to community‐wide pathogen transmission.
Network structure	The arrangement of nodes and links in an ecological network; in this study, between the lower (flower) and higher (bee visitor) trophic levels of a bipartite network. Structure could be described in the form of species‐level and community‐level metrics, such as resource overlap and connectance, respectively.
Resource overlap	Species‐level network characteristic describing the similarity of interactions between two species of the same trophic level (similarity in floral preference).
Connectance	Community‐level network characteristic describing the proportion of observed links (unique interactions) to all possible links in a network.
Competence	The ability of a host bee species to acquire and transmit a virus.
Viral load	The number of viral copies (genome equivalents) present in or on a host.
Viral exposure	The number of transmissible viral particles (virions) available at a site, calculated as: (log‐transformed key host density per m^2^ of flowers × key host viral prevalence × key host average viral load at the site level).

Bees (Anthophila) provide a valuable and important test case to explore pathogen epidemiology in multispecies communities. They comprise a rich diversity of wild and managed taxa of considerable ecological and economic importance through their pollination services (Aizen et al. 2019). However, they experience widespread declines in abundance and diversity (Potts et al. 2010; Zattara and Aizen 2021), potentially driven down by a wide range of pathogens (Goulson et al. 2015; Pluta and Paxton 2022; Potts et al. 2016). The managed honeybee ( *Apis mellifera* ) suffers elevated mortality brought on by exotic *Varroa destructor* (varroa) mites through feeding on bee brood and adults and pathogen vectoring (Traynor et al. 2020).

Wild bee species, bumblebees (*Bombus* spp.) in particular, frequently harbour deformed wing virus (DWV) and other ‘honeybee’ viruses, including black queen cell virus (BQCV) and acute bee paralysis virus (ABPV) (Fürst et al. 2014; Manley et al. 2019; Maurer et al. 2024; McMahon et al. 2015), though varroa itself is restricted to honeybees (Traynor et al. 2020). The prevailing paradigm explaining wide host‐viral associations is that of viral spillover from honeybees to wild bee species at shared flowers through the ingestion of contaminated material like pollen, nectar or faeces (Alger et al. 2019; Burnham et al. 2021; Figueroa et al. 2019). Honeybees exhibit multiple traits supporting their status as a superspreader (key host) species: perennial, highly social behaviour of colonies augments pathogen accumulation, persistence, and spread (Manley et al. 2015; Wham et al. 2024); long seasonal activity, polylecty and large foraging radius extend the reach of their infectious material across flower species (Seeley 1985); and high density on flowers increases the probability of transmission (Hung et al. 2018). While some bee‐associated viruses, such as DWV‐A, show little to no replication (e.g., in *Osmia bicornis* ; Schauer et al. 2023) or onwards transmission potential in non‐*Apis* hosts (e.g., in *Bombus terrestris* ; Tehel et al. 2022), others such as DWV‐B, BQCV and ABPV can be widespread and even more prevalent (Box 1) in wild species than in honeybees (Doublet et al. 2025; McMahon et al. 2015). It is thus likely that these viruses have alternative key hosts.

Although identification of the key hosts of a multi‐host pathogen is complex, one means of estimating their contribution to pathogen persistence is to calculate the basic reproduction number (*R*_0_; Fenton et al. 2023; Streicker et al. 2013; Viana et al. 2014; Box 1). For multi‐host pathogens, *R*_0_ must reflect the whole community of susceptible hosts that contribute to varying extents to the persistence of a pathogen (Fenton et al. 2015; Rudge et al. 2013). Due to difficulty in estimating *R*_0_ based on field data, this insightful approach goes vastly unexplored in pollinator research (but see: Figueroa et al. 2020).

Additionally, in systems where interspecific pathogen spread is strictly tied to foraging patterns, such as pollinator communities, the measurable interactions of animals with plants (flower‐visitor network structure, Box 1) are likely to alter the course of transmission (Espira et al. 2022). For example, bee species visiting the same flowers (high resource overlap; Box 1) have a higher risk of virus transmission; thus, the resource overlap with the key host might explain transmission patterns of pathogens (Maurer et al. 2024).

Yet, if the spread of a pathogen is influenced by multiple, highly competent host species (Box 1), focusing solely on a single key host may not fully capture the pathogen's transmission dynamics. In many cases, transmission is shaped by an interplay between the contributions of individual hosts and broader community‐wide properties (Page et al. 2025). Metrics such as the total density of all host species or measures of contact rates within the ecological network can therefore complement host‐specific insights by explaining additional variation in pathogen prevalence. For example, in an all‐susceptible host community, pathogen transmission is expected to be highest in well‐connected (connectance, Box 1) networks (Proesmans et al. 2021) and increase with rising host density (Anderson and May 1981). Therefore, it is also important to understand which factors influence network characteristics. For example, resource availability may change the network structure, leading to altered pathogen transmission pathways (Manley et al. 2023), regardless of whether the spread is driven by one or several key host species. Similarly, an increased density of bees can affect network structure by prompting avoidance behavior and niche partitioning (Casanelles‐Abella et al. 2023). Several studies have investigated the relationships among host species (usually honeybees), network structure, and pathogen prevalence in bee communities (Manley et al. 2023; Maurer et al. 2024; Proesmans et al. 2026). However, no empirical efforts have yet been made to rigorously identify key hosts of viral transmission.

**FIGURE 1:**
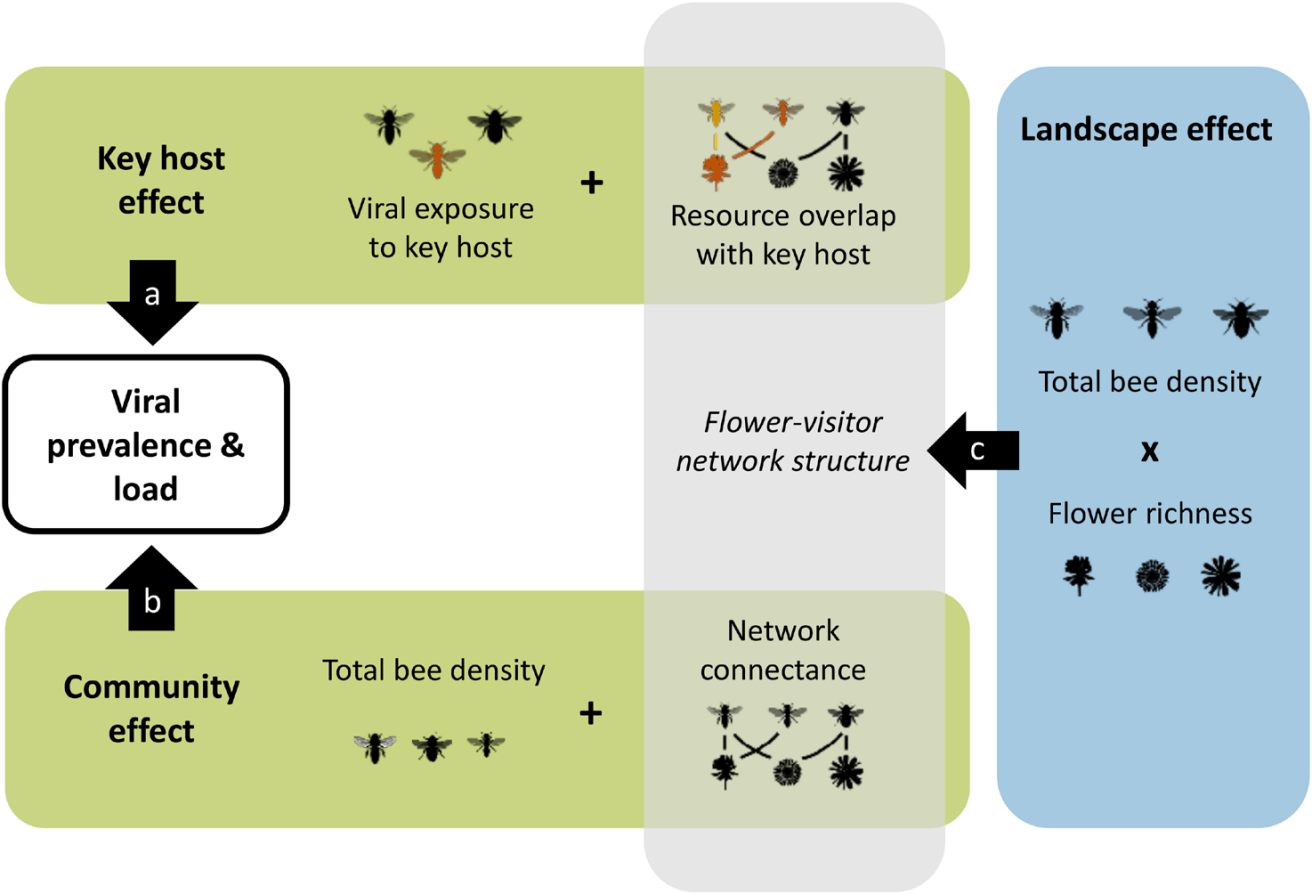
In our causal framework, the prevalence and load of viruses in bee communities (encapsulated box) can be driven by a key host (a) and community factors (b), modulated through the structure of flower‐visitor networks. Landscape‐level characteristics such as bee and flower densities can interactively affect the network structure (c), thus indirectly acting on pathogen transmission (encapsulated box). Bee silhouettes by Melissa Broussard via phylopic.org (https://creativecommons.org/licenses/by/4.0/).

Here, using field data from 48 landscape‐scale flower‐visitor networks and viral screening of 1725 individuals across 10 bee species, (i) we use mathematical modelling to identify the key host species for three viral pathogens: DWV‐B, BQCV and ABPV, by estimating the basic reproduction number for each virus–host combination (*R*_0_,_i_). Thereafter, (ii) we test how viral occurrence in the bee community is shaped by viral exposure to the identified key host, by host‐level network structure (resource overlap), and by community‐level network structure (connectance; Figure 1). Finally, (iii) we investigate how landscape‐level floral density and bee density influence flower‐visitor network structure, with potential indirect consequences for pathogen spread (Figure 1).

## 2 Materials and Methods

### 2.1 Study Design

We selected 32 agricultural study sites in southern Lower Saxony and northern Hesse, Germany (Figure S1; Table S1) varying in habitat diversity from simple landscapes dominated by conventional crop fields to diverse landscapes with various pollinator‐oriented conservation measures (Czechofsky et al. 2025; Pluta et al. 2024; Appendix S1). We used half of the sites (*n* = 16) in 2021 surveys and all sites (*n* = 32) in 2022 surveys. We collated multiple land‐use data sources (Appendix S1) and mapped habitat types using QGIS (QGIS Development Team 2018) at the 500 m radius from the center of each site. Each site was additionally ground‐truthed in both years to check the accuracy of mapping.

Though we attempted to manipulate honeybee density by supplementing sites with four or 84 additional small colonies, this did not result in a marked increase in honeybee density in the field (Figure S1). We focus on recording of in‐field densities of honeybees on flowers—rather than colony density—in our analyses (see Appendix S1).

### 2.2 Landscape‐Level Characteristics: Bee Density and Floral Resources

To create flower‐visitor networks for network structure analysis, we surveyed all sites within a 500 m radius from its centre in July 2021 and 2022. We recorded bee visitations to flowers along seven transect walks per site, each of 200 m^2^ (usually 200 × 1 m) and each 10 min long (excluding insect handling time), spread across three different habitat types in proportion to their relative abundance at a site, if present ((i) annual flower fields, (ii) organic crops and (iii) perennial semi‐natural habitats, including fallows, other conservation measures and grassy field margins; Czechofsky et al. 2025). We also calculated flower cover by visually estimating the percentage cover of open flowers in the transect areas. Plants were identified with the Flora Incognita identification app (Mäder et al. 2021). All transects were sampled once per year.

We identified bees in the field or collected specimens for identification with standard keys (Amiet et al. 2017; Mauss 1994), or by a bee taxonomy expert (Jenny Förster, Dresden). We extrapolated the density of each bee species to the 500 m radius based on pollinator abundance in the transects following Czechofsky et al. (2025), but also corrected the bee density for flower abundance (Appendix S1).

We combined the surveys (seven transect walks) from each site into one network per year and calculated network indices that might be predictive for pathogen transmission in pollinators (Proesmans et al. 2021), as they reflect the potential indirect contact of infected and susceptible individuals through flower visitation: (i) floral resource overlap for each pair of species in the network, expressed as Morisita's index using the ‘spaa’ package (Zhang 2025) and (ii) connectance of the whole network using the ‘bipartite’ package (Dormann et al. 2022). Resource overlap serves as a measure of possible contact between individuals of two species, while connectance represents the general rates of contact between all pollinators present in the network (Appendix S1).

### 2.3 Virus Data Collection

We collected female bees for viral screening in July of both years using hand‐netting within a 500 m radius at each site. In 2021, bees were sampled from 16 of the 32 sites, and in 2022 from all 32 (Table S1). Network surveys and pathogen sampling were conducted within the same 5‐day period. At each site, we collected 10 honeybees, 12–22 bumblebees ( *B. lapidarius* , *B. pascuorum* and in 2021 also *B. terrestris* ), and at least 10 other non‐*Bombus* wild bees (Table S2). Individuals were screened for three viruses (DWV‐B, BQCV and ABPV; Table S3) by qPCR, yielding presence/absence and viral load data (Box 1). Full molecular methods are provided in Appendix S1.

### 2.4 Basic Reproduction Number (*R*_0,i_) of Bee Viruses

To reveal the putative key host for each virus, we estimated the basic reproduction number (*R*_0,i_) for each host species following methodology developed by Fenton et al. (2015). This method takes into consideration the possible contribution of all host species in a network to the prevalence of a pathogen in a focal species, based on viral prevalence, host abundance, host shedding rate and resource overlap among all pairs of species. The higher the value of *R*_0,i_, the more species *i* contributes to transmission within its own population and the less it relies on cross‐species transmission to maintain viral prevalence, indicating that it may be a highly competent host. The basic reproduction number (*R*_0,i_) for each bee species is defined as (Fenton et al. 2015):

(1) $$R_{0,i}=\frac{1}{\left(1-P_{i}^{*}\right)\sum_{j=1}^{n}\left(\delta_{\mathrm{ij}}\varepsilon_{\mathrm{ij}}\theta_{\mathrm{ij}}\omega_{\mathrm{ij}}\right)},$$

where:

- P*_i_ is the viral prevalence in focal host species *i*,
- *δ*_*ij*_ = *λ*_*j*_/*λ*_*i*_ and *λ*_*i*(*j*)_ is the shedding rate of species *i (j)*,
- *ε*_*ij*_ = H_j_/*H*_*i*_ and *H*_*i*(*j*)_ is the abundance of species *i (j)*,
- *θ*_*ij*_ = P*_j_/P*_i_,
- *ω*_*ij*_ = *β*_*ij*_/*β*_*ii*_ and *β*_*ij*_ is the interspecific transmission between species *i* and *j*.
- *β*_ii_ is the intraspecific transmission within species *i*.

We used the mean species viral load per site and year as a proxy of shedding rate and the resource overlap between each pair of species per site as *β*_*ij*_. We assigned a *β*_*ii*_ value of one to solitary bees and a value of two to social bees so that social species have half the *ω*_*ij*_ values (and thus twice the *R*_0,i_ value) due to intracolony transmission (sensitivity analysis in Table S4). We do not consider the effect of varroa as an intraspecific vector of viruses in honeybee colonies, which could increase *R*_0,i_ for honeybees, because the elevated mortality connected to mite infestation will likely limit the spread of a vectored pathogen (Martin et al. 2012; Traynor et al. 2020). Owing to the targeted collection of bees for pathogen screening, sampling effort differed between bee species. To account for that uncertainty, instead of using raw prevalence data, we used model‐predicted prevalence, which skews the prevalence away from the prior based on the number of data points available for each species (Appendix S1; Table S5).

We tested the importance of putative key hosts to pathogen persistence and spread in the bee community by excluding separately the two species with highest *R*_0,i_ (see Section 3) from the models and reformulating formula (1) to calculate simulated prevalence using previously estimated *R*_0_ values (Appendix S1). A stark decrease in simulated prevalence would indicate that the key host of the virus is also important for interspecific spread of that virus.

### 2.5 Statistical Analysis

We performed our analyses using R v4.4.1 in R Studio version 2024.04.1 (R Core Team 2018). We fitted models within a Bayesian Markov chain Monte Carlo (MCMC) framework using the brms package (Bürkner 2017) as the surface for the Stan programming language (Stan Development Team 2023). We ran each model with four chains and 5000 iterations, including 2000 warmup iterations.

To test whether key host‐level and community‐level factors explain viral prevalence and load, we used generalised linear mixed models (GLMMs) with hurdle log‐normal distribution (details in Appendix S1) separately for each bee group (honeybees, bumblebees, wild bees). We used viral exposure to the key host, resource overlap with the putative key host, network connectance, the logarithm of total bee density per m^2^ of flowers and year as explanatory variables. We also used a two‐way interaction term between viral exposure and resource overlap and retained it in the model if the probability of direction of the interaction was at least 0.95 (Appendix S1). We calculated viral exposure as the product of host density (plus one), viral prevalence, and viral load to obtain the density of viral particles per m^2^ of flowers. Then, we log‐transformed the obtained viral exposure (plus one). Similarly, the logarithm of total bee density was calculated after adding one to avoid underestimation of bee density.

‘Site’ was included as a random factor in all models. In bumblebee models, we additionally included ‘Species’ as a fixed factor with three levels and, in non‐*Bombus* wild bee models, we included ‘Species’ as a random factor with 16 levels. We used weakly informative priors in all models (details in Appendix S1).

We carried out sensitivity analyses to assess the robustness of our models (Appendix S1, Tables S10 and S11). The results are reported alongside the main results.

To examine whether landscape features influence transmission dynamics, we modelled the effects of landscape‐level flower and bee densities on network structure. Connectance was modelled with a beta distribution using flower density, bee density (per m^2^ of flowers), and their interaction as predictors; the interaction was retained when pd > 0.95. Network size (sum of all nodes) was included as a covariate. Resource overlap between the two key viral hosts and other bees was modelled using LMMs with a normal distribution, as beta models failed posterior predictive checks. We used the same predictors as in the connectance models and included Site and Species as random effects because resource overlap is specific to each species–site combination.

For data handling and visualisation, we used the following packages: dplyr, tidyr, tidybayes, ggplot2, rphylopic and cowplot (Gearty and Jones 2023; Kay 2024; Wickham 2016; Wickham et al. 2023, 2024; Wilke 2024). For simplicity, we refer to the predicted probability of viral detection as the predicted prevalence. To improve visualisation of the hurdle term of the models, we inverted the probability so that 0 means 0% predicted prevalence, and 1 means 100% predicted prevalence. Results presented in the tables and text are raw estimates.

## 3 Results

We recorded 6024 interactions within 48 flower‐visitor networks across 2 years and screened 470 honeybees, 878 bumblebees and 424 non‐*Bombus* wild bees for viruses. Honeybees and three bumblebee species, the red‐tailed bumblebee *Bombus lapidarius* , buff‐tailed bee *B. terrestris* agg. and common carder bee *B. pascuorum*, occurred most frequently in networks (Table S6). The same species were also most often positive for the three screened viruses: DWV‐B, BQCV and ABPV (prevalence and loads in Table S2, Figure S2).

### 3.1 Species‐Specific *R*_0_

We calculated *R*_0,i_ for seven bee species with 2021 data and for 13 bee species with 2022 data, based on Equation (1), incorporating host abundance, viral prevalence, viral shedding rate (viral load), contact between host species (resource overlap) and accounting for sociality of a species.

**FIGURE 2:**
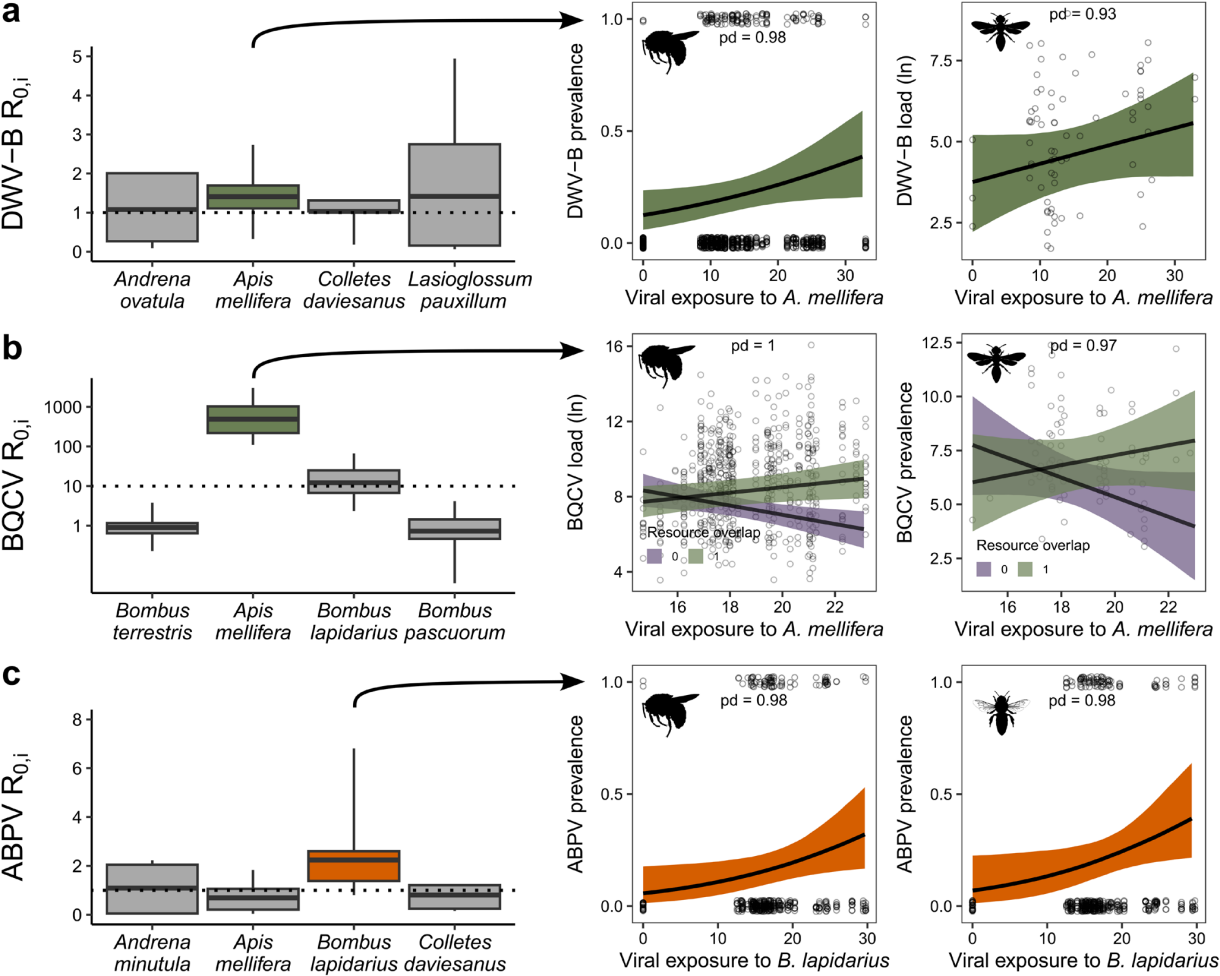
Left side: Species‐specific basic reproduction numbers (*R*_0,i_) of the four bee species with the highest *R*_0,i_ across all networks: *Andrena ovatula* (*n* = 6 networks), *Apis mellifera* (*n* = 46), *Colletes daviesanus* (*n* = 5), *Lasioglossum pauxillum* (*n* = 11), *Bombus lapidarius* (*n* = 46), *Bombus pascuorum* (*n* = 43) and *Bombus terrestris* (*n* = 15) for (a) deformed wing virus (DWV‐B), (b) black queen cell virus (BQCV) and (c) acute bee paralysis virus (ABPV). Boxplots show mean (central bar), interquartiles (coloured box), 1.5 × interquartile (whiskers) and outliers (dots). Right side: The viral exposure to the species with the highest *R*_0,i_ (i.e., the key host; *A. mellifera* for DWV‐B and BQCV, and *B. lapidarius* for ABPV) was used in statistical models to predict the detection of viruses in other bees (shown are predictions for bumblebees and wild bees for DWV‐B and BQCV and non‐ *B. lapidarius* ‐bumblebees and honeybees for ABPV). Points show raw data, and shaded areas show 95% CI. pd. = probability of direction. Probability of the hurdle term was inverted for visualisation. Bee silhouettes by Melissa Broussard via phylopic.org (https://creativecommons.org/licenses/by/4.0/).

DWV‐B had the highest *R*_0,i_ in the honeybee (*R*_0,i_ = 1.41 ∓ 0.44; mean ∓ standard deviation across all networks) and in *Lasioglossum pauxillum* (*R*_0,i_ = 1.41 ∓ 1.63); however, in *L. pauxillum* *R*_0,i_ was high only in networks where it did not share floral resources with other pollinators (Figure S3). *R*_0,i_ of BQCV was again highest in the honeybee (*R*_0,i_ = 728.74 ∓ 703.46), and also markedly higher in *B. lapidarius* (*R*_0,i_ = 17.26 ∓ 15.84) than other species (Figure 2b). *R*_0,i_ of ABPV was highest in *B. lapidarius* (*R*_0,i_ = 2.24 ∓ 1.31), followed by *A. minutula* (*R*_0,i_ = 1.09 ∓ 0.96; Figure 2c, Table S6) but, similarly to *L. pauxillum* , *R*_0,i_ for *A. minutula* was high only in networks with low resource overlap with other species (Figure S3).

**FIGURE 3:**
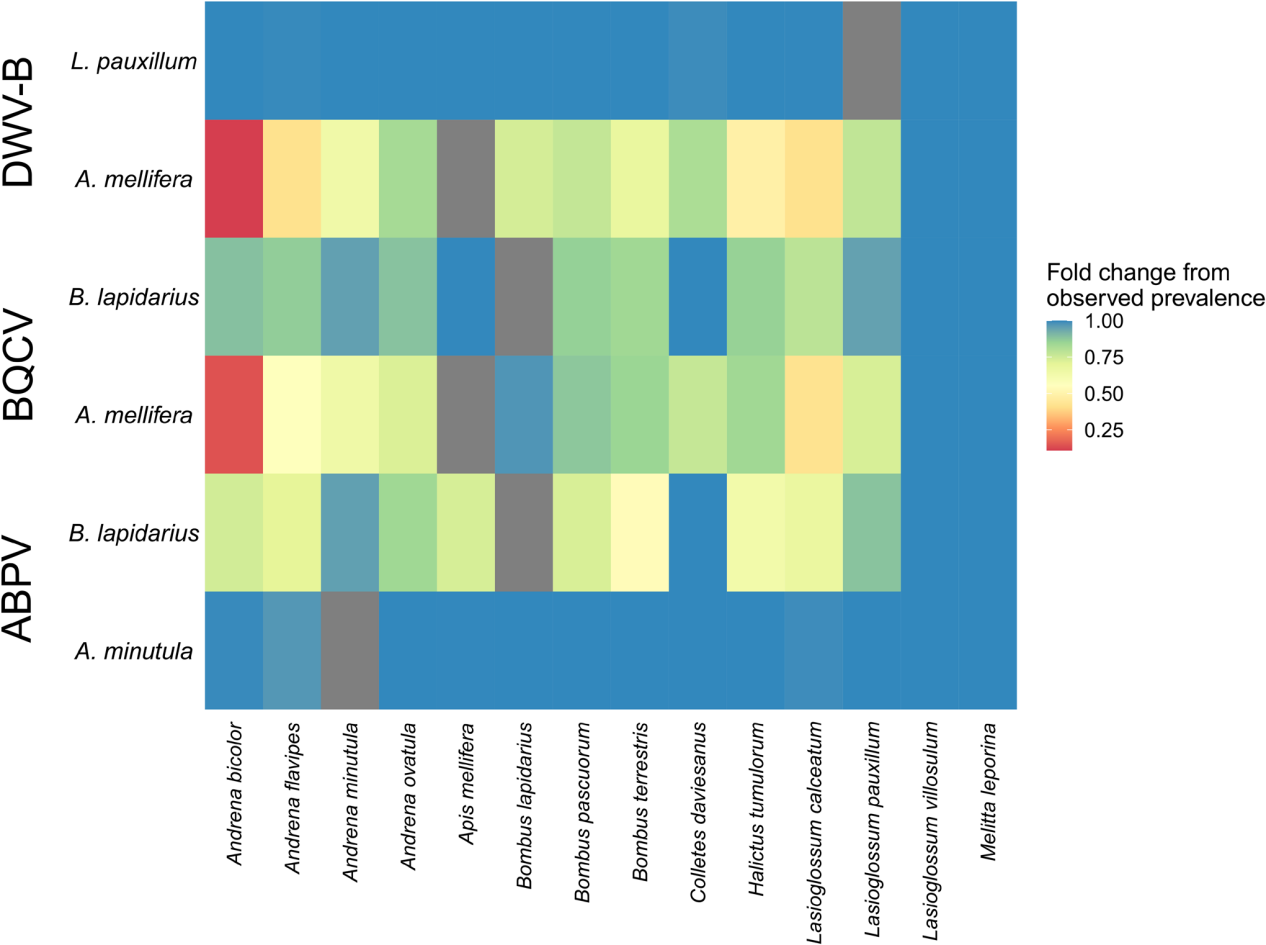
Heatmap representing fold change in simulated prevalence after theoretical removal of a key host (*y* axis), relative to the observed prevalence and adjusted for sample size. *X* axis indicates all the species present in the *R*_0_ calculation; the *Y* axis indicates which species was removed before calculation of the prevalence of the remaining species. Redder colours represent greater decrease in simulated prevalence after the removal of the key host and blue colours indicate minimal change. Grey tiles indicate the key host that has been removed.

We explored the role of key hosts by testing whether the simulated prevalence after removal of the first or second host with highest *R*_0,i_ was lower than the observed prevalence (Table S7). For all three viruses, removal of the first host (honeybee in DWV‐B and BQCV, *B. lapidarius* in ABPV), but not the second host ( *L. pauxillum* in DWV‐B, *B. lapidarius* in BQCV and *A. minutula* in ABVP) resulted in an overall decrease in prevalence in other species (Table S8, Figure 3).

### 3.2 The Effect of Key Host and Community Metrics on Viral Prevalence and Load in Communities of Bees

Because our simulations showed that each virus's primary host (honeybee for DWV‐B and BQCV, *B. lapidarius* for ABPV) was crucial for its persistence, we used these hosts to explain virus prevalence and load in bee communities. Our hurdle models show that viral exposure to honeybee was positively related to DWV‐B prevalence in bumblebees and, with lesser probability, DWV‐B load in wild bees (*β*_hurdle_ [95% highest posterior density (HPD)] = −0.36 [−0.69, −0.02], probability of direction (pd) = 0.98 and *β*_hurdle_ [95% HPD] = 0.43 [−0.14, 1.00], pd = 0.93, respectively; Figure 2a). For BQCV, viral exposure interacted with resource overlap: BQCV load increased with increasing viral exposure when resource overlap was high, but decreased when resource overlap was low in both bumblebee and wild bee models (*β*_load_ [95% HPD] = 0.37 [0.15, 0.59], pd = 1.00 and *β*_load_ [95% HPD] = 0.66 [−0.06, 1.39], pd = 0.96, respectively; Figure 2b). Viral exposure to *B. lapidarius* was also positively related to the prevalence of ABPV in other bumblebees and honeybees (*β*_hurdle_ [95% HPD] = −0.42 [−0.84, −0.04], pd = 0.98 and *β*_hurdle_ [95% HPD] = −0.34 [−0.81, 0.07], pd = 0.95, respectively; Figure 2c, Table S9).

**FIGURE 4:**
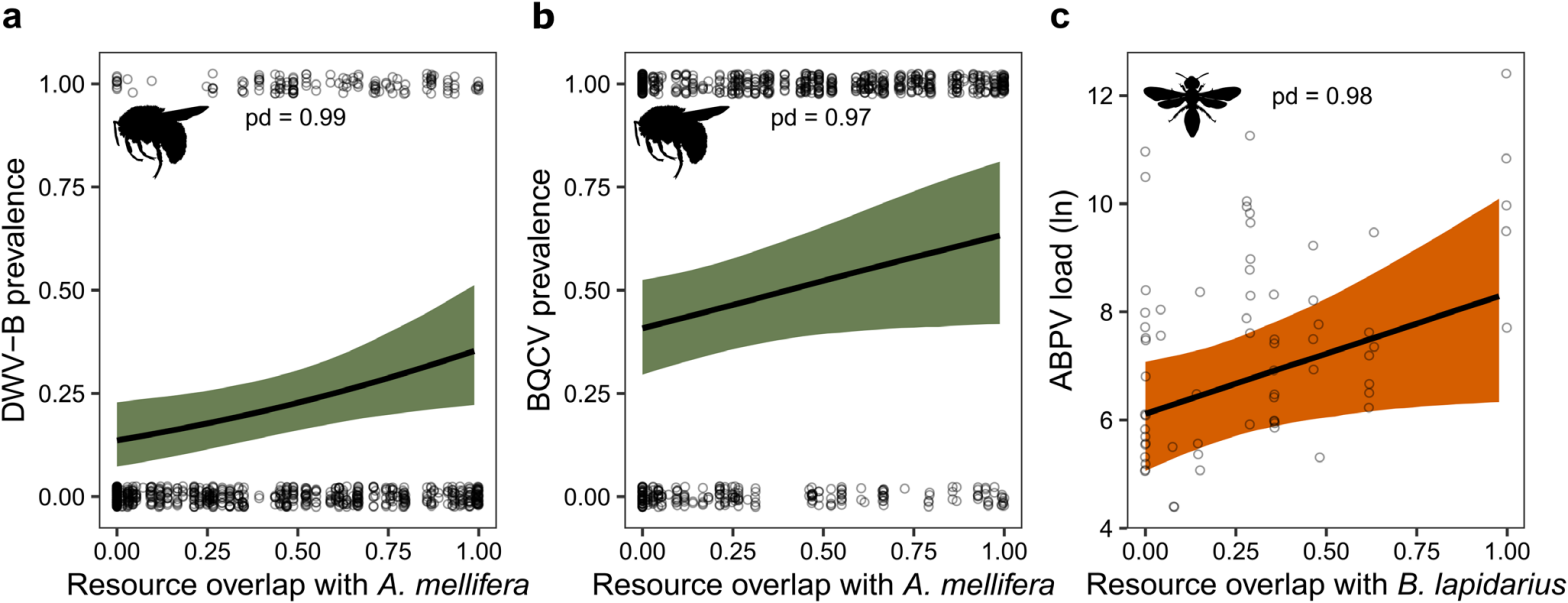
Predicted prevalence or load (viral genome equivalents) of a virus in relation to floral resource overlap with the key host (honeybee, *Apis mellifera* , in green, and red‐tailed bumblebee, *Bombus lapidarius* , in orange) for (a) DWV‐B prevalence in bumblebees, (b) DWV‐B load in bumblebees and (c) ABPV load in non‐*Bombus* wild bees. Points show raw data and shaded areas show 95% CI. pd. = probability of direction. Probability of the hurdle term was inverted for visualisation. Bee silhouettes by Melissa Broussard via phylopic.org (https://creativecommons.org/licenses/by/4.0/).

In bumblebees, resource overlap with honeybee was positively related to the prevalence of DWV‐B (*β*_hurdle_ [95% HPD] = −0.43 [−0.79, −0.08], pd. = 0.99; Figure 4a) and BQCV (*β*_hurdle_ [95% HPD] = −0.32 [−0.66, 0], pd. = 0.97; Figure 4b). Moreover, resource overlap with *B. lapidarius* was related to the load of ABPV in other wild bees (*β*_load_ [95% HPD] = 0.53 [0.01, 1.03], pd. = 0.98; Figure 4c, Table S9).

**FIGURE 5:**
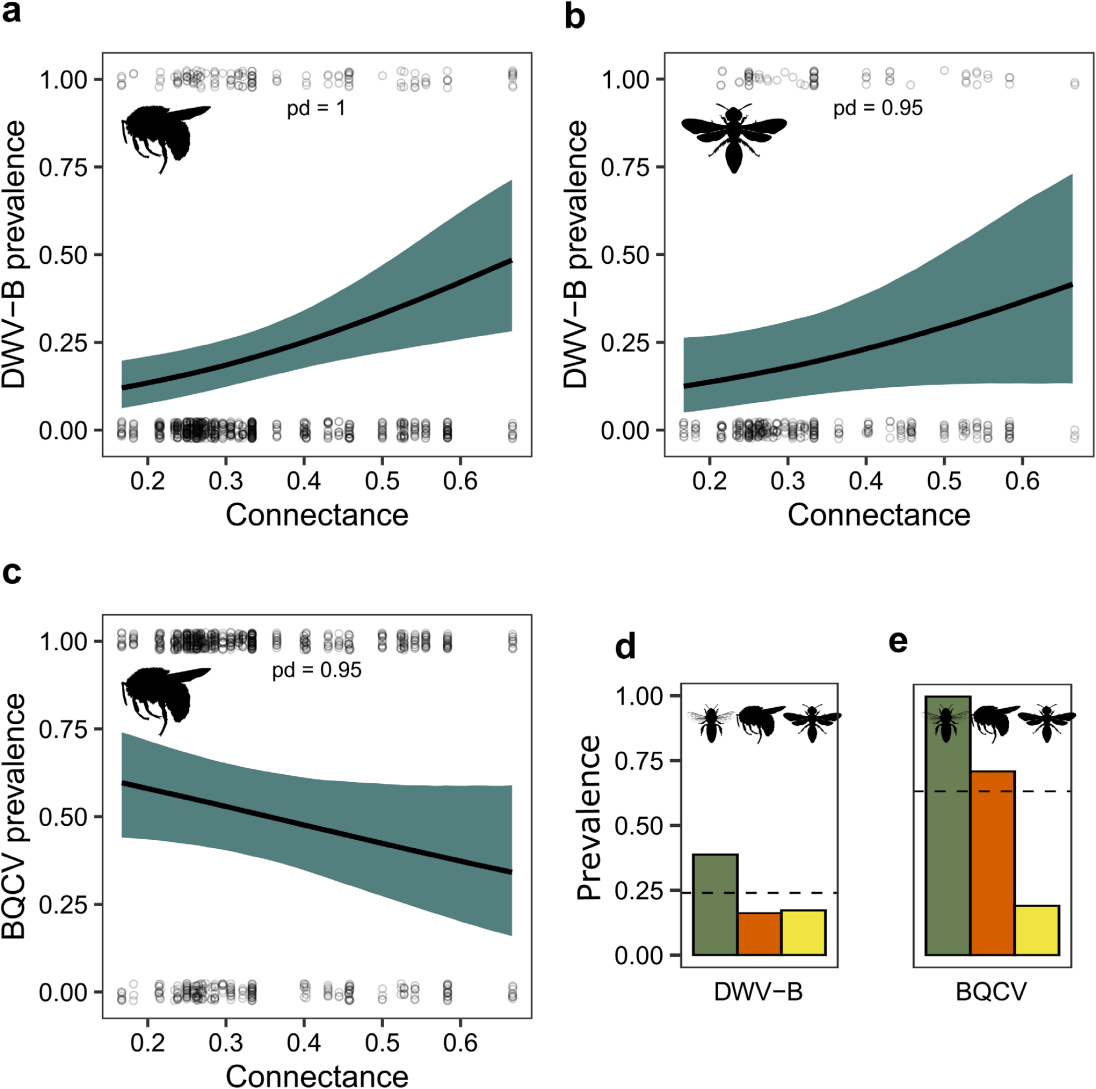
Predicted viral prevalence in relation to network connectance for DWV‐B prevalence in (a) bumblebees and (b) non‐*Bombus* wild bees and (c) BQCV in bumblebees. (d) DWV‐B and (e) BQCV prevalence in the three bee groups: dashed lines indicate the mean prevalence across all bee groups (community prevalence). Points show raw data, and shaded areas show 95% CI. pd. = probability of direction. Probability of the hurdle term was inverted for visualisation. Bee silhouettes by Melissa Broussard via phylopic.org (https://creativecommons.org/licenses/by/4.0/).

Higher connectance was related to higher prevalence of DWV‐B in bumblebees (*β*_hurdle_ [95% HPD] = −0.47 [−0.80, −0.17], pd. = 1.00; Figure 5a) and other wild bees (*β*_hurdle_ [95% HPD] = −0.38 [−0.81, 0.07], pd. = 0.95; Figure 5b), both of which had observed DWV‐B prevalence below the community mean (Figure 5d). At the same time, connectance was linked to lower prevalence of BQCV in bumblebees (*β*_hurdle_ [95% HPD] = 0.26 [−0.04, 0.56], pd. = 0.95; Figure 5c), their observed prevalence being above the community mean (Figure 5e). Neither prevalence nor load of the studied viruses was related to total bee density (Table S9).

Our sensitivity analysis including network size in models to test whether network metrics are sensitive to changes in network dimensions showed that our results on resource overlap are robust, but that connectance is strongly collinear with network size, which produces uninterpretable estimates for connectance and network size (posterior correlation = −0.83; Tables S10 and S11).

### 3.3 Network Metrics Linked to Landscape‐Level Resources

We examined the relationship between network structure and landscape‐level characteristics. Flower and bee density were not related to connectance or resource overlap with honeybees when accounting for network size (Table S12). Resource overlap with *B. lapidarius* was positively linked only to flower density (β [95% HPD] = 0.14 [0.00, 0.29], pd = 0.97; Table S12). The interaction between flower density and bee density was not likely (pd < 0.95) in any of the models.

## 4 Discussion

By quantifying *R*_0,i_ across bee species in ecological networks and modelling viral prevalence and load, we shed novel insights into the dynamics of viral pathogens and their spillover in communities of bees. In particular, we highlight the complexity of pathogen transmission dynamics in a multi‐faceted system that insect pollinators represent, and challenge the assumption of viral spillover taking place exclusively from honeybees to wild bees—community‐wide viral prevalence can be driven not only by honeybees but also by wild hosts such as *B. lapidarius* . Moreover, we show that the theoretical removal of a key host from the community may lead to a decrease in viral prevalence, but only in species that share common floral resources, underpinning the importance of generalist species in viral spread within bee communities.

Our key host removal simulations highlight that high *R*_0,i_ of a host species does not necessarily translate into high interspecific spread of the pathogen. For example, removing the secondary hosts *L. pauxillum* and *A. minutula* did not reduce prevalence in other pollinators, despite high *R*_0,i_ of DWV‐B and ABPV in these species, respectively, because of low floral resource overlap with other pollinators. In contrast, the removal of the honeybee from DWV‐B and BQCV models, and *B. lapidarius* from the ABPV model, two polylectic species, led to a marked decrease in prevalence across the whole community. Similarly, viral transmission in species that do not share floral resources with a key host might rely on secondary hosts for viral acquisition. Due to the limited research on viral susceptibility in wild bees, we cannot draw conclusions about the competence of *L. pauxillum* and *A. minutula* to DWV‐B and ABPV—viral *R*_0,i_ for these species might be grossly overestimated if their actual competence and shedding rate are low. However, previous studies have reported the presence and high prevalence of these viruses in both genera (Doublet et al. 2025; Maurer et al. 2024; Yañez et al. 2020). The growing abundance of field‐collected data on viruses in bees could benefit tremendously from controlled experiments on competence (Page et al. 2025).

Our statistical models showed a clear link between viral exposure to the key host and the prevalence and load of viruses in bees. This finding is in line with previous research (Alger et al. 2018; Maurer et al. 2024; Proesmans et al. 2026; Tuerlings et al. 2022) reporting a relationship between viruses in honeybees and wild bees. However, our results also highlight another key host of viral spread in pollinators: *B. lapidarius* . The ecology of *B. lapidarius* is similar to that of honeybees: both species are social, visit a wide range of flowers, and are abundant in nature, including our study sites. While the most efficient route of intraspecific transmission for many viruses (DWV‐B and ABPV in our study) in honeybees is the varroa mite vector (Yañez et al. 2020), *B. lapidarius* and other wild bees do not have any known biological or mechanical vectors of viruses. Nevertheless, *B. lapidarius* had a consistently higher prevalence of ABPV than honeybees. The mortality associated with injection‐induced ABPV infection, which mimics transmission through varroa mites, is very high in honeybees (Bailey et al. 1963; Bailey and Gibbs 1964; Bailey and Woods 1974), which seems to effectively keep ABPV prevalence low in *A. mellifera* (Martin 2001; Sumpter and Martin 2004). Experimental studies suggest, however, that bumblebees suffer similarly from ABPV when injected (Bailey and Gibbs 1964), although no study to date has examined the effect of ABPV when ingested, which is the most likely route of transmission in wild bees (Yañez et al. 2020).

Key hosts are typically defined by high abundance, prevalence, or shedding rate (Streicker et al. 2013). In flower‐mediated pollinator pathogens (Figueroa et al. 2020), floral visitation patterns further shape transmission, so pathogen spread also depends on the structure and interactions of the whole pollinator community. We show that increasing resource overlap with the key host, which we use as a proxy of contact rate (bees visiting the same flowers), is linked to increased prevalence and load of viruses. This result aligns with previous research that also took into consideration species‐specific resource overlap (Maurer et al. 2024; Proesmans et al. 2026), rather than average resource overlap across the whole network (Manley et al. 2023). The latter approach might dilute the impact of key host(s) when specialist species are present in the network. It is possible, however, that viral stability on flowers plays a role, for example, certain flowers produce secondary compounds that might inactivate viral particles impeding transmission. Increased resource overlap on these flowers might reduce viral load, as viruses may not be able to replicate. The role of viral persistence on flower surfaces has yet to be explored in comprehensive studies. Interestingly, for BQCV we observe that the relationship between viral exposure and BQCV load in bumblebees and wild bees is mediated by resource overlap, where the load increases only when resource overlap with honeybee is high. It suggests that the prevalence of BQCV in honeybees might be already high enough to saturate the infection rate, but sharing of resources remains critical for successful transmission. This is further highlighted by the positive relationship between resource overlap with honeybee and prevalence of BQCV in bumblebees.

Beyond the influence of key hosts, we found that the community‐level structure of flower‐visitor networks might also shape pathogen prevalence in bees. Network connectance was positively associated with DWV‐B prevalence in bumblebees and other wild bees, but negatively associated with BQCV prevalence. This pattern is consistent with Figueroa et al. (2020), who showed that greater connectance reduces variance in prevalence, raising it in species with initially low prevalence and lowering it in species with high prevalence. Because connectance is strongly correlated with network size, however, we cannot fully separate its effect from changes in network dimensionality. Nevertheless, the high *R*_0_,_i_ of viruses in species with limited resource overlap, such as DWV‐B in *L. pauxillum* , suggests that community‐level processes, not just overlap with the key host, contribute to pathogen spread.

The absence of a relationship between total bee density and virus prevalence or load suggests that pathogen spread may be driven primarily by the key host, consistent with other systems (McCallum et al. 2017). However, because bee density often correlates with species richness (Marini et al. 2025) and richness and density have opposing effects on transmission—richness reducing infection risk (Fearon and Tibbetts 2021) and density increasing it for density‐dependent pathogens (Johnson et al. 2024), drawing clear conclusions remains challenging.

Our calculation of *R*_0_ assumed that all bee species can acquire virus from the environment and transmit it onwards, with the shedding rate proportional to their infection intensity (viral load). This necessarily simplifies the system because we lack knowledge on the competence of wild bees to viruses and their shedding rates (but see Burnham et al. 2021). While ABPV was found to replicate in many wild bee species (Doublet et al. 2025), little is known of the susceptibility of wild bees to DWV‐B and BQCV (but see: Li et al. 2011; Peng et al. 2011). Streicher et al. (2024) showed that, while *B. terrestris* excretes DWV‐B particles when fed the virus, the viral load in faeces is much lower compared to that in faeces of *A. mellifera* . Furthermore, Dalmon et al. (2021) and Mckeown et al. (2025) showed that some wild bee species might harbour different viral genotypes than that of honeybees, which could impair interspecific transmission. Because most experimental studies focus on honeybee and bumblebees, findings for other wild bee species remain far more uncertain. Pollinators may also adjust their behaviour by detecting recently visited or contaminated flowers, as shown for the parasite *Crithidia bombi* (Fouks and Lattorff 2011), which could further alter exposure risk. These factors underscore additional environmental and behavioural processes that may influence viral dynamics in pollinator communities. Another shortcoming of our study is the lack of information on viral baseline at our sites and in our honeybee colonies before their placement in the field. Despite mitigation steps to ensure low pathogen burden in the introduced hives, we cannot robustly assess what the direct impact is of the honeybee colonies on the viral landscape in bee communities.

Overall, we found no strong evidence that landscape‐level floral and bee density shape flower‐visitor network structure. However, resource overlap with the key host *B. lapidarius* increased at sites with high flower density, suggesting that resource availability may influence certain network parameters with potential implications for pathogen transmission.

Our results highlight the complexity of viral spread in communities where viral transmission is tied to foraging for food resources. We show that, despite the lack of experimental data, key host species can be identified empirically, rather than assumed. At the same time, we point out that, in multispecies host communities, removal of the key host might not lead to the desired reduction in pathogen prevalence when interaction breadth or resource overlap of remaining competent species is wide; exploration of community‐wide characteristics might provide additional insights into host‐pathogen dynamics. Our study contributes to the field of EIDs research, wherein identification and control of key hosts is nevertheless of utmost importance for the well‐being of wildlife, livestock and humans.

## Author Contributions

Robert J. Paxton, Catrin Westphal and Annika L. Hass designed the study and acquired funding. Patrycja Pluta and Annika L. Hass conceived the analysis. Patrycja Pluta and Kathrin Czechofsky collected the data. Patrycja Pluta performed the laboratory work. Patrycja Pluta performed the analysis. Patrycja Pluta wrote the manuscript with contributions from all authors.

## Funding

The study was supported by funds from the Federal Ministry of Food and Agriculture (Bundesministerium für Ernährung und Landwirtschaft; BMEL) based on a decision of the parliament of the Federal Republic of Germany via the Federal Office for Agriculture and Food (Bundesanstalt für Landwirtschaft und Ernährung; BLE) under the Federal Programme for Organic Farming (Bundesprogramm Ökologischer Landbau; BÖL) (grant numbers 2819OE115 and 2819OE156). Catrin Westphal is grateful for funding from the German Research Foundation (Deutsche Forschungsgemeinschaft; DFG)–Project numbers: 405945293 and 493487387.

## Supporting information


**Figure S1:** Study sites and honeybee density.
**Figure S2:** Average observed prevalence and load of viruses across bee groups.
**Figure S3:** The relationship between resource overlap and *R*_0_.

**Table S1:** Location of study sites and number of samples from each bee group collected for viral screening.
**Table S2:** Number of individuals of each species collected and successfully screened for viruses throughout the study, number of sites at which the species was collencted, and the average prevalence and load of the viruses. DWV‐B, deformed wing virus genotype B; BQCV, black queen cell virus; ABPV, acute bee paralysis virus.
**Table S3:** Primer details used in the study.
**Table S4:** Sensitivity analysis of the sociality parameter effect in *R*_0,i_ modelling. Increasing parameter for social species increases *R*_0,i_ but the relative positions of host species do not change. *p* = sociality parameter (intraspecific transmission multiplier). *p* = 1 assumes no effect of sociality. *R*_0,i_ is averaged across all sites and years, that is, across the 48 networks. DWV‐B, deformed wing virus type B; BQCV, black queen cell virus; ABPV, acute bee paralysis virus.
**Table S5:** Adjusted prevalence estimation. Observed (raw) presence/absence data were used to estimate the adjusted prevalence (model predicted, based on the number of samples available) per site, year and species. Table shows average prevalence across all sites and years.
**Table S6:** Average basic reproduction number for each virus and bee species R0i across the networks and the number of networks in which the species was recorded (max = 48).
**Table S7:** Comparison of the average prevalence of viruses in bee species: adjusted prevalence (based on the observed prevalence), prevalence after theoretical removal of the first key host, and prevalence after theoretical removal of the second key host.
**Table S8:** Bayes factors of key host removal and intercept only models. Model 1 is compared with Model 2; a Bayes factor above 1 indicated support in favour of Model 1, a value below 1 indicates lack of support for Model 1.
**Table S9:** Results of the hurdle models of viral prevalence and load. Bold terms indicate probability of direction (pd) equal to or higher than 0.95. CI, credible interval. ESS, effective sample size.
**Table S10:** Sensitivity analysis of hurdle models. Sum of nodes is added as a covariate to every model to control for network size. Bold terms indicate probability of direction (pd) equal to or higher than 0.95. CI, credible interval; ESS, effective sample size.
**Table S11:** Sensitivity analysis of hurdle models removing species at sites where the resource overlap with the key host was not recorded. Bold terms indicate probability of direction (pd) equal to or higher than 0.95. CI, credible interval; ESS, effective sample size.
**Table S12:** Results of the community‐level models. Bold terms indicate probability of direction (pd) equal to or higher than 0.95. CI, credible interval; ESS, effective sample size.

**Appendix S1:** ele70327‐sup‐0003‐Appendix1.docx.

## Data Availability

Data and code available at: https://doi.org/10.6084/m9.figshare.30069595. Code additionally available at https://github.com/papluta/Pathogens‐Networks.

## References

[ele70327-bib-0001] Aizen, M. A. , S. Aguiar , J. C. Biesmeijer , et al. 2019. “Global Agricultural Productivity Is Threatened by Increasing Pollinator Dependence Without a Parallel Increase in Crop Diversification.” Global Change Biology 25: 3516–3527. 31293015 10.1111/gcb.14736 PMC6852307

[ele70327-bib-0002] Alger, S. A. , P. Alexander Burnham , H. F. Boncristiani , and A. K. Brody . 2018. “RNA Virus Spillover From Managed Honeybees ( *Apis mellifera* ) to Wild Bumblebees (Bombus spp.).” PLoS One 14: e0217822. 10.1371/journal.pone.0217822 PMC6594593 31242222

[ele70327-bib-0003] Alger, S. A. , P. A. Burnham , and A. K. Brody . 2019. “Flowers as Viral Hot Spots: Honey Bees ( *Apis mellifera* ) Unevenly Deposit Viruses Across Plant Species.” PLoS One 14: e0221800. 31532764 10.1371/journal.pone.0221800 PMC6750573

[ele70327-bib-0004] Amiet, F. , A. Müller , and C. Praz . 2017. “Apidae 1: Allgemeiner Teil, Gattungen, Apis, Bombus. Fauna Helvetica 29, 185 pp. Centre Suisse de Cartographie de la Faune (CSCF) and Swiss Entomological Society (SEG).”

[ele70327-bib-0005] Anderson, R. M. , and R. M. May . 1981. “The Population Dynamics of Microparasites and Their Invertebrate Hosts.” Philos. Trans. R. Soc. London. B, Biol. Sci 291: 451–524. 10.1098/rstb.2014.0307 PMC4360116 25750231

[ele70327-bib-0007] Bailey, L. , A. J. Gibbs , and R. D. Woods . 1963. “Two Viruses From Adult Honey Bees ( *Apis mellifera* Linnaeus).” Virology 21: 390–395. 14081363 10.1016/0042-6822(63)90200-9

[ele70327-bib-0006] Bailey, L. , and A. J. Gibbs . 1964. “Acute Infection of Bees With Paralysis Virus.” Journal of Invertebrate Pathology 6: 395–407.

[ele70327-bib-0008] Bailey, L. , and R. D. Woods . 1974. “Three Previously Undescribed Viruses From the Honey Bee.” Journal of General Virology 25: 175–186. 4215869 10.1099/0022-1317-25-2-175

[ele70327-bib-0009] Becker, D. J. , A. D. Washburne , C. L. Faust , E. A. Mordecai , and R. K. Plowright . 2019. “The Problem of Scale in the Prediction and Management of Pathogen Spillover.” Philosophical Transactions of the Royal Society B 374: 1–9. 10.1098/rstb.2019.0224 PMC6711304 31401958

[ele70327-bib-0010] Bürkner, P. C. 2017. “Brms: An R Package for Bayesian Multilevel Models Using Stan.” Journal of Statistical Software 80: 1–8.

[ele70327-bib-0011] Burnham, P. A. , S. A. Alger , B. Case , H. Boncristiani , L. Hébert‐Dufresne , and A. K. Brody . 2021. “Flowers as Dirty Doorknobs: Deformed Wing Virus Transmitted Between Apis Mellifera and *Bombus impatiens* Through Shared Flowers.” Journal of Applied Ecology 58: 2065–2074.

[ele70327-bib-0012] Casanelles‐Abella, J. , S. Fontana , B. Fournier , D. Frey , and M. Moretti . 2023. “Low Resource Availability Drives Feeding Niche Partitioning Between Wild Bees and Honeybees in a European City.” Ecological Applications 33: e2727. 36054537 10.1002/eap.2727 PMC10077915

[ele70327-bib-0013] Czechofsky, K. , C. Westphal , R. J. Paxton , and A. L. Hass . 2025. “Landscape‐Level Synergistic and Antagonistic Effects Among Conservation Measures Drive Wild Bee Densities and Species Richness.” Journal of Applied Ecology 62: 1706–1717.

[ele70327-bib-0014] Dalmon, A. , V. Diévart , M. Thomasson , et al. 2021. “Possible Spillover of Pathogens Between Bee Communities Foraging on the Same Floral Resource.” Insects 12: 122. 33573084 10.3390/insects12020122 PMC7911050

[ele70327-bib-0015] Daszak, P. , A. A. Cunningham , and A. D. Hyatt . 2000. “Emerging Infectious Diseases of Wildlife–Threats to Biodiversity and Human Health.” Science 287: 443–449. 10642539 10.1126/science.287.5452.443

[ele70327-bib-0016] Dormann, C. F. , J. Fruend , B. Gruber , et al. 2022. “Visualising Bipartite Networks and Calculating Some (Ecological) Indices.”

[ele70327-bib-0017] Doublet, V. , T. D. Doyle , C. Carvell , M. J. F. Brown , and L. Wilfert . 2025. “Host Ecology and Phylogeny Shape the Temporal Dynamics of Social Bee Viromes.” Nature Communications 16: 1–11. 10.1038/s41467-025-57314-7 PMC11882784 40044660

[ele70327-bib-0018] Espira, L. M. , A. F. Brouwer , B. A. Han , J. Foufopoulos , and J. N. S. Eisenberg . 2022. “Dilution of Epidemic Potential of Environmentally Transmitted Infectious Diseases for Species With Partially Overlapping Habitats.” American Naturalist 199: E43–E56. 10.1086/717413 PMC9136953 35077275

[ele70327-bib-0019] Fearon, M. L. , and E. A. Tibbetts . 2021. “Pollinator Community Species Richness Dilutes Prevalence of Multiple Viruses Within Multiple Host Species.” Ecology 102: e03305. 33571384 10.1002/ecy.3305

[ele70327-bib-0020] Fenton, A. , D. G. Streicker , O. L. Petchey , and A. B. Pedersen . 2015. “Are All Hosts Created Equal? Partitioning Host Species Contributions to Parasite Persistence in Multihost Communities.” American Naturalist 186: 610–622. 10.1086/683173 PMC6542667 26655774

[ele70327-bib-0021] Fenton, A. , S. M. Withenshaw , G. Devevey , A. Morris , D. Erazo , and A. B. Pedersen . 2023. “Experimental Assessment of Cross‐Species Transmission in a Natural Multihost–Multivector–Multipathogen Community.” Proceedings of the Royal Society B: Biological Sciences 290: 20231900. 10.1098/rspb.2023.1900 PMC10646469 37964529

[ele70327-bib-0022] Figueroa, L. L. , M. Blinder , C. Grincavitch , et al. 2019. “Bee Pathogen Transmission Dynamics: Deposition, Persistence and Acquisition on Flowers.” Proceedings of the Royal Society B: Biological Sciences 286: 20190603. 10.1098/rspb.2019.0603 PMC6545085 31138075

[ele70327-bib-0023] Figueroa, L. L. , H. Grab , W. H. Ng , et al. 2020. “Landscape Simplification Shapes Pathogen Prevalence in Plant‐Pollinator Networks.” Ecology Letters 23: 1212–1222. 32347001 10.1111/ele.13521 PMC7340580

[ele70327-bib-0024] Fouks, B. , and H. M. G. Lattorff . 2011. “Recognition and Avoidance of Contaminated Flowers by Foraging Bumblebees ( *Bombus terrestris* ).” PLoS One 6: e26328. 22039462 10.1371/journal.pone.0026328 PMC3200320

[ele70327-bib-0025] Fürst, M. A. , D. P. McMahon , J. L. Osborne , R. J. Paxton , and M. J. F. Brown . 2014. “Disease Associations Between Honeybees and Bumblebees as a Threat to Wild Pollinators.” Nature 506: 364–366. 24553241 10.1038/nature12977 PMC3985068

[ele70327-bib-0026] Gearty, W. , and L. A. Jones . 2023. “Rphylopic: An R Package for Fetching, Transforming, and Visualising PhyloPic Silhouettes.” Methods in Ecology and Evolution 14: 2700–2708.

[ele70327-bib-0027] Goulson, D. , E. Nicholls , C. Botías , and E. L. Rotheray . 2015. “Bee Declines Driven by Combined Stress From Parasites, Pesticides, and Lack of Flowers.” Science 347: 1255957. 25721506 10.1126/science.1255957

[ele70327-bib-0028] Hung, K.‐L. J. , J. M. Kingston , M. Albrecht , D. A. Holway , and J. R. Kohn . 2018. “The Worldwide Importance of Honey Bees as Pollinators in Natural Habitats.” Proceedings of the Royal Society B: Biological Sciences 285: 20172140. 10.1098/rspb.2017.2140 PMC5784195 29321298

[ele70327-bib-0029] Johnson, P. T. J. , T. E. Stewart Merrill , A. D. Dean , and A. Fenton . 2024. “Diverging Effects of Host Density and Richness Across Biological Scales Drive Diversity‐Disease Outcomes.” Nature Communications 15: 1–11. 10.1038/s41467-024-46091-4 PMC10908850 38431719

[ele70327-bib-0030] Kay, M. 2024. “Tidybayes: Tidy Data and Geoms for Bayesian Models.” Package Version 3.0.6.

[ele70327-bib-0031] Li, J. , W. Peng , J. Wu , J. P. Strange , H. Boncristiani , and Y. Chen . 2011. “Cross‐Species Infection of Deformed Wing Virus Poses a New Threat to Pollinator Conservation.” Journal of Economic Entomology 104: 732–739. 21735887 10.1603/ec10355

[ele70327-bib-0032] Mäder, P. , D. Boho , M. Rzanny , et al. 2021. “The Flora Incognita App–Interactive Plant Species Identification.” Methods in Ecology and Evolution 12: 1335–1342.

[ele70327-bib-0033] Mahon, M. B. , A. Sack , O. A. Aleuy , et al. 2024. “A Meta‐Analysis on Global Change Drivers and the Risk of Infectious Disease.” Nature 629: 830–836. 38720068 10.1038/s41586-024-07380-6

[ele70327-bib-0034] Manley, R. , M. Boots , and L. Wilfert . 2015. “REVIEW: Emerging Viral Disease Risk to Pollinating Insects: Ecological, Evolutionary and Anthropogenic Factors.” Journal of Applied Ecology 52: 331–340. 25954053 10.1111/1365-2664.12385 PMC4415536

[ele70327-bib-0035] Manley, R. , V. Doublet , O. N. Wright , et al. 2023. “Conservation Measures or Hotspots of Disease Transmission? Agri‐Environment Schemes Can Reduce Disease Prevalence in Pollinator Communities.” Philosophical Transactions of the Royal Society B 378: 20220004. 10.1098/rstb.2022.0004 PMC9900712 36744563

[ele70327-bib-0036] Manley, R. , B. Temperton , T. Doyle , et al. 2019. “Knock‐On Community Impacts of a Novel Vector: Spillover of Emerging DWV‐B From Varroa ‐Infested Honeybees to Wild Bumblebees.” Ecology Letters 22: ele.13323. 10.1111/ele.13323 PMC6852581 31190366

[ele70327-bib-0037] Marini, L. , E. Gazzea , M. Albrecht , et al. 2025. “Using Total Abundance as a Proxy for Wild Bee Species Richness: A Practical Tool for Non‐Experts.” Journal of Applied Ecology 62: 3065–3077.

[ele70327-bib-0038] Martin, S. J. 2001. “The Role of Varroa and Viral Pathogens in the Collapse of Honeybee Colonies: A Modelling Approach.” Journal of Applied Ecology 38: 1082–1093.

[ele70327-bib-0039] Martin, S. J. , A. C. Highfield , L. Brettell , et al. 2012. “Global Honey Bee Viral Landscape Altered by a Parasitic Mite.” Science 336: 1304–1306. 22679096 10.1126/science.1220941

[ele70327-bib-0040] Maurer, C. , A. Schauer , O. Yañez , et al. 2024. “Species Traits, Landscape Quality and Floral Resource Overlap With Honeybees Determine Virus Transmission in Plant–Pollinator Networks.” Nature Ecology & Evolution 8: 2239–2251. 39367259 10.1038/s41559-024-02555-w PMC11618065

[ele70327-bib-0077] Mauss, V. 1994. Bestimmungsschlüssel für Hummeln (5th ed.). Deutscher Jugendbund für Naturbeobachtung, Justus‐Strandes‐Weg 14, 22337 Hamburg, Germany.

[ele70327-bib-0042] McCallum, H. , A. Fenton , P. J. Hudson , et al. 2017. “Breaking Beta: Deconstructing the Parasite Transmission Function.” Philosophical Transactions of the Royal Society B 372: 20160084. 10.1098/rstb.2016.0084 PMC5352811 28289252

[ele70327-bib-0043] Mckeown, D. A. , E. Evans , J. Helgen , et al. 2025. “Distinct Virome Compositions and Lack of Viral Diversi Fi Cation Indicate That Viral Spillover Is a Dead‐End Between the Western Honey Bee and the Common Eastern Bumblebee.” Communications Biology 8: 926. 40523966 10.1038/s42003-025-08351-x PMC12170865

[ele70327-bib-0044] McMahon, D. P. , M. A. Fürst , J. Caspar , P. Theodorou , M. J. F. Brown , and R. J. Paxton . 2015. “A Sting in the Spit: Widespread Cross‐Infection of Multiple RNA Viruses Across Wild and Managed Bees.” Journal of Animal Ecology 84: 615–624. 25646973 10.1111/1365-2656.12345 PMC4832299

[ele70327-bib-0045] Page, M. L. , J. K. Davis , S. K. Glasser , et al. 2025. “Mechanisms and Consequences of Plant–Pollinator–Pathogen Interactions.” Annual Review of Ecology, Evolution, and Systematics 56: 53–72.

[ele70327-bib-0046] Peng, W. , J. Li , H. Boncristiani , J. P. Strange , M. Hamilton , and Y. Chen . 2011. “Host Range Expansion of Honey Bee Black Queen Cell Virus in the Bumble Bee, *Bombus huntii* .” Apidologie 42: 650–658.

[ele70327-bib-0047] Pluta, P. , K. Czechofsky , A. Hass , et al. 2024. “Organic Farming and Annual Flower Strips Reduce Parasite Prevalence in Honeybees and Boost Colony Growth in Agricultural Landscapes.” Journal of Applied Ecology 61: 2146–2156.

[ele70327-bib-0048] Pluta, P. , and J. R. Paxton . 2022. “Assessing the Impact of Disease on Pollinators.” In Promoting Pollination and Pollinators in Farming, edited by P. G. Kevan and S. W. Chan . Burleigh Dodds Science Publishing.

[ele70327-bib-0049] Potts, S. G. , J. C. Biesmeijer , C. Kremen , P. Neumann , O. Schweiger , and W. E. Kunin . 2010. “Global Pollinator Declines: Trends, Impacts and Drivers.” Trends in Ecology & Evolution 25: 345–353. 20188434 10.1016/j.tree.2010.01.007

[ele70327-bib-0050] Potts, S. G. , V. Imperatriz‐Fonseca , H. T. Ngo , et al. 2016. “Safeguarding Pollinators and Their Values to Human Well‐Being.” Nature 540: 220–229. 27894123 10.1038/nature20588

[ele70327-bib-0051] Power, A. G. , and C. E. Mitchell . 2004. “Pathogen Spillover in Disease Epidemics.” American Naturalist 164: S79–S89. 10.1086/424610 15540144

[ele70327-bib-0052] Proesmans, W. , C. Alaux , M. Albrecht , et al. 2026. “Drivers of Viral Prevalence in Landscape‐Scale Pollinator Networks Across Europe: Honey Bee Viral Density, Niche Overlap With This Reservoir Host and Network Architecture.” Ecology Letters 21: e70309. 10.1111/ele.70309 PMC12767914 41489984

[ele70327-bib-0053] Proesmans, W. , M. Albrecht , A. Gajda , et al. 2021. “Pathways for Novel Epidemiology: Plant–Pollinator–Pathogen Networks and Global Change.” Trends in Ecology & Evolution 36: 623–636. 33865639 10.1016/j.tree.2021.03.006

[ele70327-bib-0054] QGIS Development Team . 2018. “QGIS Geographic Information System. Open Source Geospatial Found. Proj.”

[ele70327-bib-0055] R Core Team . 2018. “A Language and Environment for Statistical Computing. R Found. Stat. Comput.”

[ele70327-bib-0056] Rigaud, T. , M. J. Perrot‐Minnot , and M. J. F. Brown . 2010. “Parasite and Host Assemblages: Embracing the Reality Will Improve Our Knowledge of Parasite Transmission and Virulence.” Proceedings of the Royal Society B: Biological Sciences 277: 3693–3702. 10.1098/rspb.2010.1163 PMC2992712 20667874

[ele70327-bib-0057] Rudge, J. W. , J. P. Webster , D. B. Lu , T. P. Wang , G. R. Fang , and M. G. Basáñez . 2013. “Identifying Host Species Driving Transmission of Schistosomiasis Japonica, a Multihost Parasite System, in China.” Proceedings of the National Academy of Sciences of the United States of America 110: 11457–11462. 23798418 10.1073/pnas.1221509110 PMC3710859

[ele70327-bib-0058] Schauer, A. , N. Bianco , O. Yañez , A. Brown , M. Albrecht , and P. Neumann . 2023. “Deformed Wing Virus Prevalence in Solitary Bees Put to the Test: An Experimental Transmission Study.” Frontiers in Ecology and Evolution 11: 1122304.

[ele70327-bib-0059] Seeley, T. D. 1985. Honeybee Ecology: A Study of Adaptation in Social Life. Princeton University Press.

[ele70327-bib-0060] Stan Development Team . 2023. “Stan Modeling Language.”

[ele70327-bib-0061] Streicher, T. , P. Brinker , S. Tragust , and R. J. Paxton . 2024. “Host Barriers Limit Viral Spread in a Spillover Host: A Study of Deformed Wing Virus in the Bumblebee *Bombus terrestris* .” Viruses 16: 607. 38675948 10.3390/v16040607 PMC11053533

[ele70327-bib-0062] Streicker, D. G. , A. Fenton , and A. B. Pedersen . 2013. “Differential Sources of Host Species Heterogeneity Influence the Transmission and Control of Multihost Parasites.” Ecology Letters 16: 975–984. 23714379 10.1111/ele.12122 PMC4282463

[ele70327-bib-0063] Sumpter, D. J. T. , and S. J. Martin . 2004. “The Dynamics of Virus Epidemics in Varroa ‐Infested Honey Bee Colonies.” Journal of Animal Ecology 73: 51–63.

[ele70327-bib-0064] Tehel, A. , T. Streicher , S. Tragust , and R. J. Paxton . 2022. “Experimental Cross Species Transmission of a Major Viral Pathogen in Bees Is Predominantly From Honeybees to Bumblebees.” Proceedings of the Royal Society B 289: 20212255. 35168401 10.1098/rspb.2021.2255 PMC8848241

[ele70327-bib-0065] Traynor, K. S. , F. Mondet , J. R. de Miranda , et al. 2020. “ *Varroa destructor* : A Complex Parasite, Crippling Honey Bees Worldwide.” Trends in Parasitology 36: 592–606. 32456963 10.1016/j.pt.2020.04.004

[ele70327-bib-0066] Tuerlings, T. , L. Buydens , G. Smagghe , and N. Piot . 2022. “The Impact of Mass‐Flowering Crops on Bee Pathogen Dynamics.” International Journal of Parasitology: Parasites and Wildlife 18: 135–147. 10.1016/j.ijppaw.2022.05.001 PMC9108762 35586790

[ele70327-bib-0067] Viana, M. , S. Cleaveland , J. Matthiopoulos , et al. 2015. “Dynamics of a Morbillivirus at the Domestic‐Wildlife Interface: Canine Distemper Virus in Domestic Dogs and Lions.” Proceedings of the National Academy of Sciences of the United States of America 112: 1464–1469. 25605919 10.1073/pnas.1411623112 PMC4321234

[ele70327-bib-0068] Viana, M. , R. Mancy , R. Biek , et al. 2014. “Assembling Evidence for Identifying Reservoirs of Infection.” Trends in Ecology & Evolution 29: 270–279. 24726345 10.1016/j.tree.2014.03.002 PMC4007595

[ele70327-bib-0069] Wham, B. E. , E. C. McCormick , C. M. Carr , et al. 2024. “Comparison of Seasonal Viral Prevalence Supports Honey Bees as Potential Spring Pathogen Reservoirs for Bumble Bees.” Ecosphere 15: e4883.

[ele70327-bib-0070] Wickham, H. 2016. “ggplot2: Elegant Graphics for Data Analysis.” R Package Version 3.5.

[ele70327-bib-0071] Wickham, H. , R. François , L. Henry , K. Müller , and D. Vaughan . 2023. “Dplyr: A Grammar of Data Manipulation.” R Package Version 1.1.4.

[ele70327-bib-0072] Wickham, H. , D. Vaughan , and M. Girlich . 2024. “Tidyr: Tidy Messy Data.” R Package Version 1.3.1.

[ele70327-bib-0073] Wilke, C. O. 2024. “Cowplot: Streamlined Plot Theme and Plot Annotations for “ggplot2”.” R Package Version 1.1.3.

[ele70327-bib-0074] Yañez, O. , N. Piot , A. Dalmon , et al. 2020. “Bee Viruses: Routes of Infection in Hymenoptera.” Frontiers in Microbiology 11: 943. 32547504 10.3389/fmicb.2020.00943 PMC7270585

[ele70327-bib-0075] Zattara, E. E. , and M. A. Aizen . 2021. “Worldwide Occurrence Records Suggest a Global Decline in Bee Species Richness.” One Earth 4: 114–123.

[ele70327-bib-0076] Zhang, J. 2025. “Package “spaa”. Species Association Analysis.” R Package Version 0.2.2.

