# Supplementary material for: Multiple Key Hosts and Network Structure Shape Viral Prevalence Across Multispecies Communities of Bees
Source: Ecol Lett. 2026 Jan 28;29(2):e70327. doi: 10.1111/ele.70327 (PMC12853010; doi:10.1111/ele.70327)
Supplement: Supplementary file 1 — Figure S1: Study sites and honeybee density. Figure S2: Average observed prevalence and load of viruses across bee groups. Figure S3: The relationship between resource overlap and R 0. [file ELE-29-0-s002.docx]

# Supporting information

**Key hosts and ecological network structure shape viral prevalence across multispecies communities of bees**

**Content:**

Supplementary Figure 1: Study sites and honeybee density.

Supplementary Figure 2: Average observed prevalence and load of viruses across bee groups.

Supplementary Figure 3: The relationship between resource overlap and R_0_.


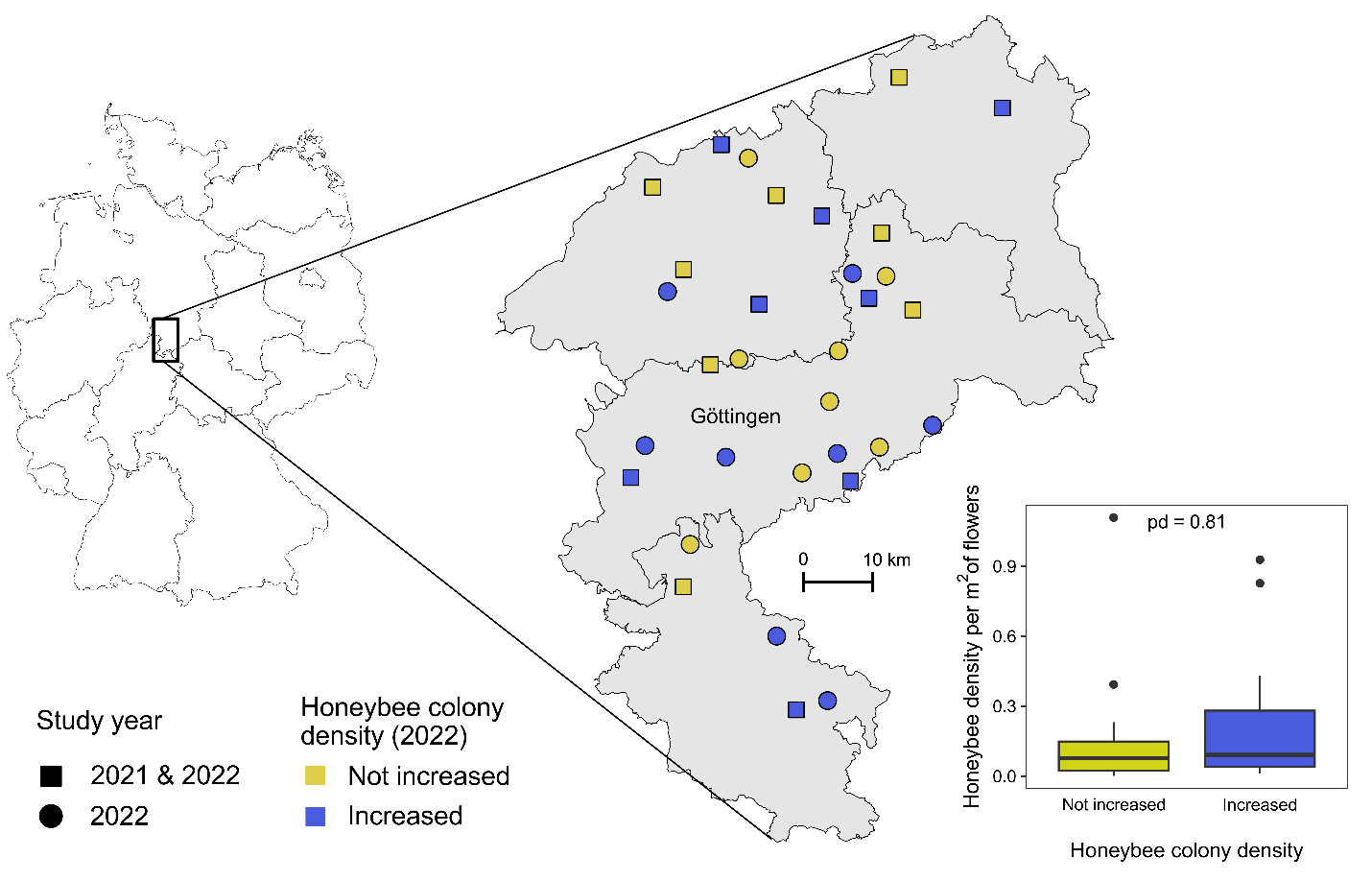


**Figure S1.** Study sites visited in both years (2021, 2022; squares) or only in 2022 (circles) in relation to the city of Göttingen, Germany. In 2021 all sites had a low (not increased) honeybee colony density. Yellow - low honeybee colony density sites in 2022; blue - high honeybee colony density sites in 2022. The boxplot showing median honeybee density per m^2^ of flowers, as observed during bee surveys at low and high colony density sites in 2022.


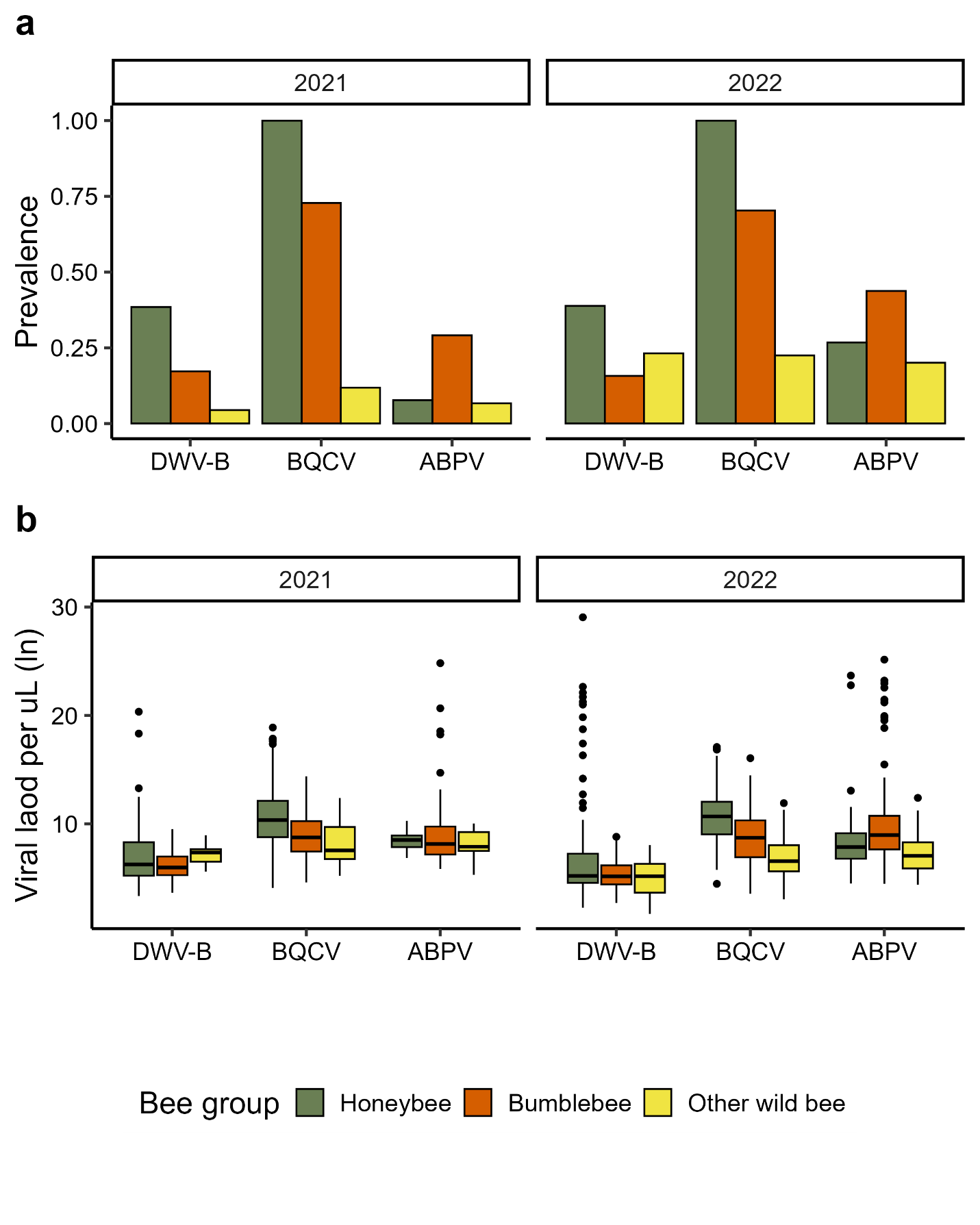


**Figure S2.** **a.** Observed viral prevalence and **b.** viral load (genome equivalents) in bee groups across the two years of the study. DWV-B – deformed wing virus genotype B, BQCV – black queen cell virus, ABPV = acute bee paralysis virus.


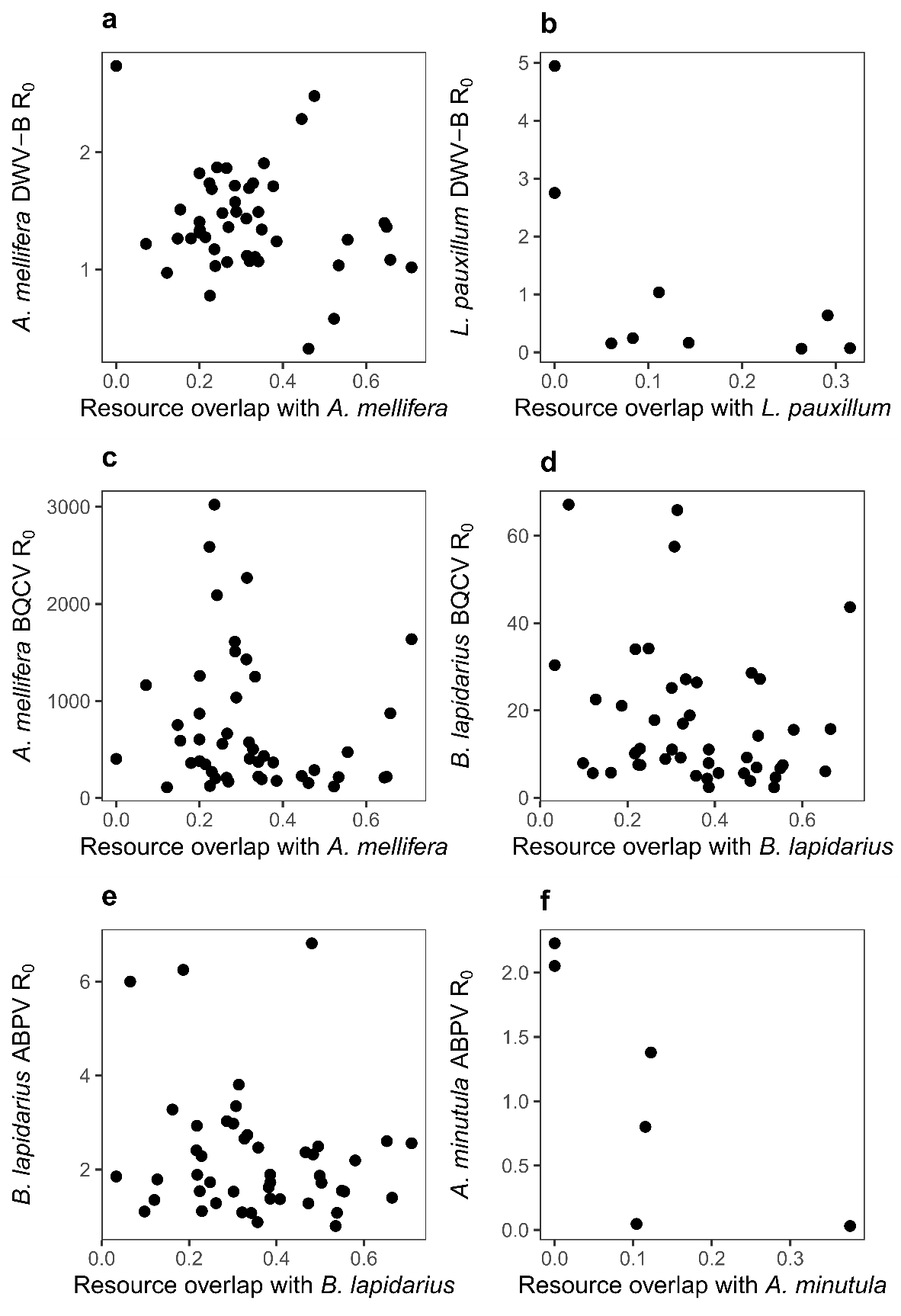


**Figure S3.** The relationship between species-specific R_0_ and resource overlap in two hosts with highest R_0_ for each virus. Each point represents one network in which the host was present. Resource overlap was calculated as an average resource overlap of that species with all other species present in the network.
