# Supplementary material for: Multiple Key Hosts and Network Structure Shape Viral Prevalence Across Multispecies Communities of Bees
Source: Ecol Lett. 2026 Jan 28;29(2):e70327. doi: 10.1111/ele.70327 (PMC12853010; doi:10.1111/ele.70327)
Supplement: Supplementary file 3 — Appendix S1: ele70327‐sup‐0003‐Appendix1.docx. [file ELE-29-0-s003.docx]

Supporting information
Appendix with extended methods

**Key hosts and ecological network structure shape viral prevalence across multispecies communities of bees**

**Content:**

1. Study design
2. Extrapolation of bee density
3. Viral screening
4. Deriving model-predicted prevalence from observed prevalence
5. Estimating the importance of putative key hosts
6. Extended statistical analysis

# Study design

- 1. Landscape

We based our habitat classification on multiple sources: Copernicus Land Monitoring Service; Official Topographical-Cartographic Information System (ATKIS: www.lgln.niedersachsen.de), the LEA portal of Lower Saxony (https://sla.niedersachsen.de/landentwicklung/LEA/) and the InVeKoS (Integriertes Verwaltungs- und Kontrollsystem) data in Hesse. We used IACS data (Integrated Administration and Control System) to map the Agri-Environment Schemes (AES). We included the following habitat types in our mapping: conventional farming, organic farming, fallow, conventional grassland, organic grassland, plantation, forest, urban area, and conservation measures incorporated within AES: annual flower field, perennial flower field, mixed flower field, grassy field margin and semi-natural habitat (SNH). SNH consisted of perennial, low-intensity features that did not fit into other categories, such as hedges and tree groups, and non-grassland grassy areas. The most common conventional crops were winter wheat (*Triticum aestivum*), winter barley (*Hordeum vulgare*), sugar beet (*Beta vulgaris*), and rapeseed (*Brassica napus*). The most common organic crops were winter wheat, field clover (*Trifolium campestre*), and field beans (*Vicia faba*). Most common flowering plant genera recorded were: *Trifolium, Vicia* and *Cirsium* in organic fields and all SNH except for flower fields, *Cirsium, Verbascum* and *Hypericum* in perennial flower fields, and *Phacelia, Cirsium* and *Melilotus* in annual flower fields.

At the time of sampling, we did not identify any blooming mass-flowering conventional crops but we did record mass-flowering organic crops across the sites that covered between 0 and 2.4% of the area in 2021 and 0 and 1.9 % in 2022.

- 1. Experimental honey bee colonies

In order to manipulate honeybee density at the sites, in May of each study year, we installed small experimental honeybee colonies at the centre of each study landscape (four colonies at each of the n=16 sites in 2021, and either four or 84 colonies at each of the n=32 sites in 2022). Placement of the colonies in 2022 was arranged such that eight of the sites used in 2021 had four colonies and the remaining eight had 84 to allow comparability across years. From the remaining 16 sites introduced in 2022, half had four hives and other half 84, assigned to achieve comparability in the landscape complexity across treatment groups. The hives were removed in March the following year. Each hive contained four frames, including two brood frames and two empty frames intended for nectar and pollen storage.

All colonies originated from approximately 100 source colonies managed by the same beekeeper. To standardize pathogen prevalence, genetic background, and overall health across experimental colonies, we created mixed pools of brood frames. Brood frames with their associated workers were randomly assigned to groups of 27 frames, and these groups were kept intact for a week to allow the adult bees from different colonies to intermingle. We then assembled each experimental colony by randomly selecting two brood frames, each drawn from a different pool, ensuring that workers originated from multiple source colonies. The source colonies had been treated against the ectoparasitic and exotic mite *Varroa destructor* (varroa) during the preceding winter. All colonies appeared healthy on inspection and, because the homogenization procedure was designed to equalize pathogen levels across hives, we did not screen colonies for micro-parasites at the outset of the experiment.

- 1. Field surveys

At each site we carried out seven 200m^2^ (usually 200 x 1 m) transect walks in different habitat types to collect information on the flower-pollinator interactions and pollinator abundance. All seven transect walks at each site were carried out within one day between 9 AM and 6 PM in favorable conditions (minimum 15°C, no precipitation, low wind speed). For the purpose of the study, we categorized *Bombus* and non-*Bombus* wild bees separately because bumblebees generally have higher prevalence of bee-associated viruses than other wild bees (Manley *et al.* 2023; Maurer *et al.* 2024; Fig. S2 in supplementary figures). Additionally, bumblebees are social and other wild bees mostly solitary; sociality may markedly elevate intraspecific pathogen transmission. We aggregated *Bombus terrestris* and *B. lucorum* as these two species are difficult to differentiate in the field, although our molecular barcoding of *B. terrestris* agg. samples for pathogen screening revealed only *B. terrestris*. Consequently, we refer to *B. terrestris* as *B. terrestris* agg. when referring to transect walks results and *B. terrestris* when referring to pathogen screening results.

- 1. Network metrics

We chose two network metrics that represent two scales of pathogen transmission: floral resource overlap (at the species or key host scale), and connectance (at the community scale). We chose floral resource overlap, calculated as Morisita index, as the species-level metric as it informs about the similarity of interactions between each pair of species in the network; it is tied directly to pathogen transmission on flowers, as transmission is more likely between species that visit the same flowers (Maurer *et al.* 2024). We chose connectance as the community-level metric as it represents a less direct way of transmission (pollinators serving as external vectors of viruses, transporting infectious material with e.g. pollen onto other flowers), and also describes transmission well in systems with many susceptible species and low heterogeneity in competence (Figueroa *et al.* 2020; Proesmans *et al.* 2021). Moreover, connectance is related to other network metrics commonly used in epidemiology, such as nestedness, which in turn correlate with many other network metrics, such as specialisation (H2’), interaction evenness, and others (Fig A1 in our study; Song *et al.* 2017). For that reason as well as to avoid inflating type I error rates (Blüthgen & Staab 2024), we abstain from testing multiple network metrics such as nestedness, H2’ or interaction evenness that may be collectively associated with community-wide transmission.


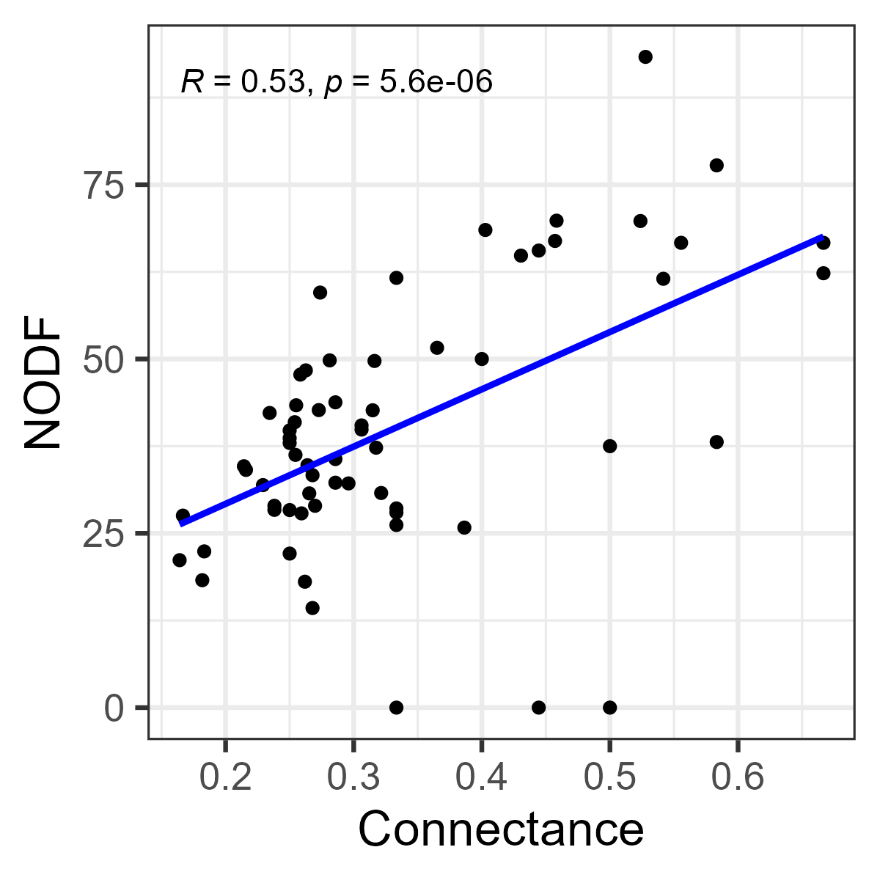


Fig A1. The relationship between nestedness (NODF) and connectance, based on the 48 flower-visitor networks in our study.

When comparing networks, one has to take into account the inherent dependence of network structure on its size (Dormann et al. 2009; Henriksen et al. 2019) and possible confounders such as differing sampling effort/completeness, i.e., larger networks are often undersampled due to difficulties in collecting information on all interactions (Fründ *et al.* 2016). Connectance is strongly tied to network size, as the number of possible links usually increases disproportionately faster than the number of realised links (Blüthgen & Staab 2024; Jordano 1987; Figure A2a). This relationship also holds for other network size metrics, such as (log) geometric mean of flowers and pollinators.

Resource overlap is also dependent on network size; however, the effect on pairwise resource overlap will most likely differ consistently between generalist and specialist species. In our study, we focus on resource overlap with two highly polylectic (generalist) pollinators (see Results; *Apis mellifera* and *Bombus lapidarius*), thus the effect of network size on our measure of resource overlap is negligible (Figure A2b).


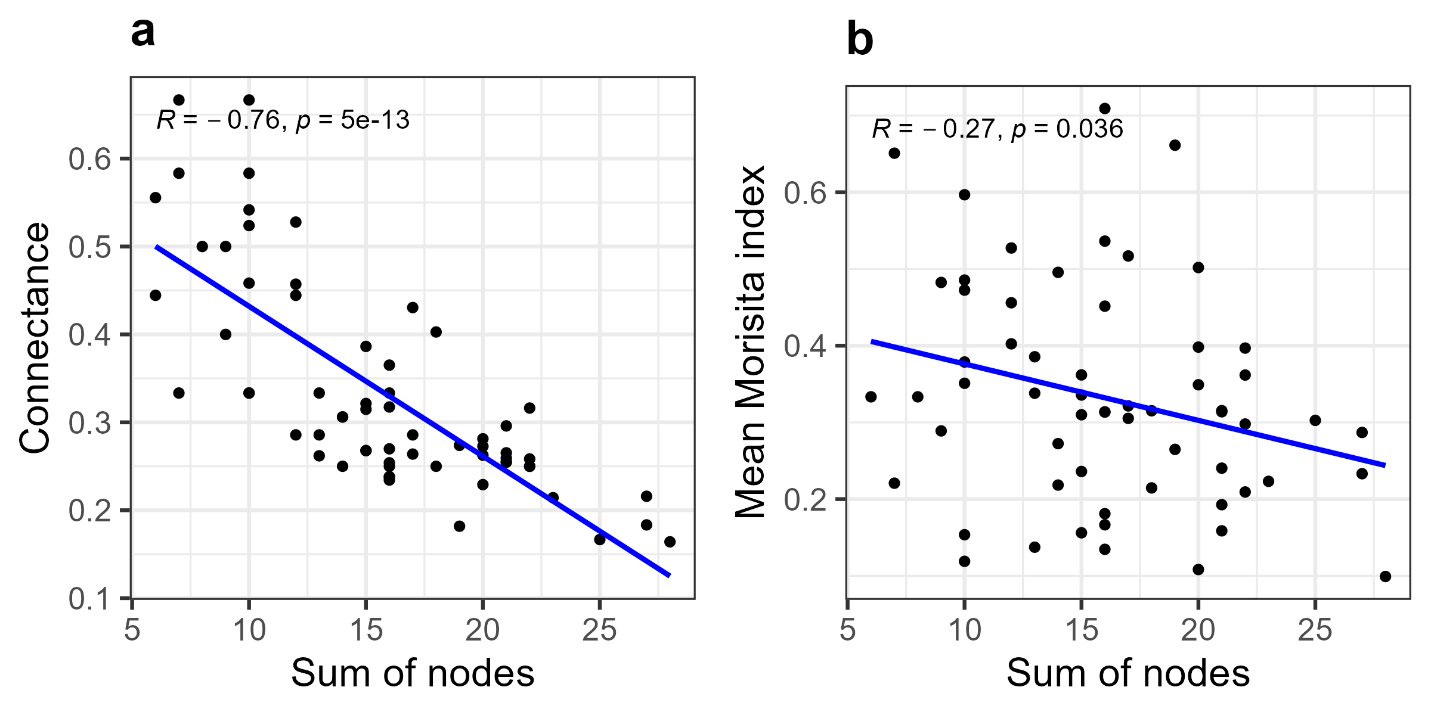


Fig A2. Relationships between network size (total number of nodes) and (a) connectance, and (b) resource overlap (Morisita index) with the focal species of the study: *Apis mellifera* and *Bombus lapidarius*, averaged per network.

A common technique to account for network size and completeness is the use of null models (Dormann *et al.* 2009; as used in: Figueroa *et al.* 2020; Manley *et al.* 2023) and the calculation of z-scores, which assess how much the observed value differs from the expected value, calculated on random networks with the same number of nodes and links. Negative z-scores indicate less structure than expected, and positive z-scores indicate more structure than expected (Dormann *et al.* 2009). These models often constrain the number of links and thus connectance (but see: Patefield 1981) and are unfit to provide unbiased estimates for link-density based metrics. Additionally, the standard deviation calculated based on the null models decreases as the network size increases, which allows for greater z-scores in large networks, introducing another bias to the metric (Song *et al.* 2017).

Another approach of correcting for network size and sampling completeness is to add network size metrics as covariates (sum of all nodes, geometric mean of higher and lower-level species, linkage density) to regression models. This method allows for estimation of untransformed estimates, which are often easier to interpret, while holding network size constant. However, network metrics, such as connectance, are often strongly colinear with network size, which produces biased estimates and prevents interpretation of the results of the colinear parameters.

We acknowledge that there is a lack of scientific consensus when trying to account for network size in network comparisons (Blüthgen & Staab 2024), and that, for example, z-scores do not necessarily correct for network size or sampling completeness. Consequently, we decided use raw values of resource overlap and connectance in our models with cautious interpretation of the connectance results (see Results, Fig. 5) and an additional sensitivity analysis using network size as a covariate (see Table S10).

# Extrapolation of bee density

To extrapolate the density of bees to the whole 500 m radius of the sampling area, we calculated the average bee abundance and flower cover per habitat type for each site, and then extrapolated the values to the 500 m radius by multiplying respectively bee abundance and flower cover with the total area of each habitat type (see Czechofsky et al. 2025 for details). If a habitat type was present at a site but the bee abundance or flower cover was not estimated for it, we used a global average of that metric. We then summed the bee abundance per species from all habitats per site and divided it by the summed area of flowers (m^2^ of flower-covered habitat per site) to obtain the density of each bee species per m^2^ of flowers within the 500 m radius. The standardization of bee density per m^2^ of flowers allowed us to derive a more realistic measure of viral exposure exerted by a key host, as bee density is tightly linked to flower availability (Beyer *et al.* 2021). Consequently, increasing the density of infected individuals in the system inevitably increases viral exposure and the risk of infection for other susceptible hosts (Stewart Merrill *et al.* 2022).

# Viral screening

Bees were collected into microfuge tubes and immediately put on dry ice, then transferred in the lab into a -80°C freezer until further processing. Pollen and pollen residues were removed from individuals with the use of forceps to minimize the detection of viral material from the exterior of a bee. Each bee was homogenized in 175-1000 µL 1 % beta-mercaptoethanol-RLT buffer mix, depending on the size of the bee, using a TissueLyzer LT (Qiagen, Hilden, Germany) and a 5 mm stainless steel bead. The homogenate was centrifuged and 100 µL was used to extract RNA using an RNeasy Mini Kit (Qiagen, Hilden, Germany) in a Qiacube robot (Qiagen, Hilden, Germany) following the manufacturer’s instructions. The quality of RNA was assessed using an Epoch Microplate Spectrophotometer (BioTek, Waldbronn, Germany), after which 800 ng of RNA was used to synthesize cDNA using MLV-RT reverse transcriptase (Promega, Mannheim, Germany) following the manufacturer’s instructions.

We screened cDNA for three common bee viral targets (DWV-B, BQCV and ABPV) by qPCR using a QuantStudio3 qPCR machine (Applied Biosystems/Thermofisher Scientific, Massachusetts, USA) and SYBRgreen Sensimix (Bioline, Luckenwalde, Germany). Each plate contained two negative controls (RNAse-free water as template) and one positive control (purified PCR product). We ran the samples as two technical replicates for 40 cycles, using the mean Cq value for further analysis. If the standard deviation (SD) of the technical replicates exceeded 1, we repeated the reaction as a triplicate and used the mean of the two closest Cq values. To assess the quality of the RNA extraction, cDNA synthesis and qPCR, we amplified 𝞫-actin genes for honeybee and bumblebee, and 28S (ribosomal subunit 28) gene for non-*Bombus* wild bees as internal reference (primers and qPCR protocols in Table S3). We estimated cut-off values for reference genes by calculating the mean Cq + 2 x SD for each gene. Samples exceeding this threshold were excluded from analysis (n = 53 samples). A sample was considered positive for a virus if at least two replicates showed successful amplification and their melting curve profiles aligned with the positive control. We quantified absolute viral load using external standard curves unique to each target. To create the curves, we used a purified PCR product with a known amount of viral genome equivalents (GE), measured with Qubit (Thermo Fisher Scientific, Massachusetts, USA) in a series of 7-8 logarithmic dilutions. Standard curves ranged from 10^9^ to 10^2^ (Cq = 11 - 36, β = -3.51, E = 93 %, *r*^2^ = 0.99) for ABPV, 10^9^ to 10^2^ (Cq = 7 – 36, β = -3.52, E = 92 %, *r*^2^ = 0.99) for BQCV, and 10^9^ to 10^1^ (Cq = 7 - 33, β = -3.50, E = 93 %, *r*^2^ = 0.99) for DWV-B. We verified the accuracy of the external standard curves by rerunning a subset of 60 samples with a new standard curve on the same plate; Cq values were within one SD. We standardised viral titres to GE/µL of bee homogenate for statistical analysis so as to account for bee body size.

We identified honeybees morphologically. To identify wild bees screened for viruses, we sequenced a highly conserved mitochondrial gene region (partial COI, the universal animal barcode) using the residual DNA present in RNA extracts (primer details in Table S3). Non-bees and unidentifiable samples were excluded from the analysis.

# Deriving model-predicted prevalence from observed prevalence

To account for the uncertainty in estimating the viral prevalence in rare/undersampled species, we used model-derived prevalence estimates that take into consideration the number of individuals available in the analysis. We ran models separately for each virus using the ‘brms’ package (Bürkner 2017) with a Bernoulli distribution and presence/absence data. For each bee species, we used normally distributed priors with a standard deviation of 3 and a mean calculated from the average prevalence across all sites, with the exception of non-*Bombus* wild bees, for which we used the mean prevalence of all non-*Bombus* wild bees because of the low number of samples for each separate species. We used ‘Site’ as a random factor. In this way, no bee species had a viral prevalence equal to 0 or to 1 (Table S5). In the case of bee species for which the observed prevalence was 0 and thus no viral load could be calculated, we used a very low load of 100 genome equivalents (GE) in the R_0_ calculation. We excluded bee species that were not recorded in either pathogen data or transect data at the same site, which reduced the number of species for the calculation of R_0_. However, the low abundance of these species makes it unlikely that they contributed significantly to the spread of pathogens, with the exception of *B. terrestris* agg. that was sampled in 2021 for pathogen screening, but not in 2022.

# Estimating the importance of putative key hosts

To investigate the importance of key host species to the persistence of a virus in the community of bees, we reframed the formula used to calculate R_0,i_, solving this time for viral prevalence, and excluding first the putative key host and then the second most likely key host from the dataset. This method reflects the predicted pathogen prevalence in host species after the culling of the key host (Fenton *et al.* 2015). A sharp decrease in the predicted prevalence suggests high importance of the removed host to the transmission of the pathogen. We fitted a prevalence model with the type of prevalence (before or after host removal) as an explanatory variable, and also included covariates: year as fixed factor and site and species as random factors. We then fitted a null model containing only covariates. We estimated the relative evidence for the effect of removal of the host species by computing the Bayes factors of the prevalence model relative to the null model. A Bayes factor greater than 1 provides evidence in favor of the prevalence model over the null model. We repeated this for the three viruses and the first and second key host separately using the brms package (Bürkner 2017). We used normal priors (0,1) for fixed factors, exponential (1) for standard deviation of random factors, and a beta distribution in all models.

# Extended statistical analysis

We analysed viral prevalence and viral load in bees using GLMMs with a hurdle log-normal distribution. The hurdle component estimates the probability that a sample contained detectable virus (Bernoulli-distributed), whereas the log-normal component models viral load only for positive samples. We fitted separate models for honeybees, bumblebees, and non-*Bombus* wild bees, and analysed each virus (DWV-B, BQCV, ABPV) independently. To avoid simultaneously treating a species as both predictor and response, we excluded the putative key host of each virus from analyses of that virus.

Across all hurdle models, we incorporated the same set of explanatory variables, as follows. We quantified viral exposure to the key host as the product of host density plus one, viral prevalence, and viral load, which yields the density of viral particles per m^2^ of flowers; we then log-transformed this quantity after adding one to accommodate zeros. We also included each species’ resource overlap with the key host, network connectance, log-transformed total bee density per m^2^ of flowers, and year. We used a two-way interaction term between viral exposure and resource overlap and retained it in the model if the probability of direction of the interaction was greater than 0.95 (see below). We used the average resource overlap with the key hosts at sites, where the interaction was not recorded (within the same year, if available, or global mean); as a sensitivity analysis (Table S11) showed that it did not generally affect the results connected with resource overlap. At two sites we did not collect the key host of ABPV for viral screening, but transect walks confirmed its presence. We have used average viral prevalence and load in that key host. In bumblebee models, species entered as a fixed factor with three levels, whereas in wild-bee models it entered as a random factor with sixteen levels. All models included site as a random intercept.

To assess how landscape-level characteristics may indirectly influence pathogen transmission by altering network structure, we modelled variation in both connectance and resource overlap. We modelled connectance with a beta distribution and included flower density per m^2^, total bee density per m^2^ of flowers, their two-way interaction as predictors and network size. To model resource overlap between each key host and other bees, we fitted LMMs with a normal distribution and used the same explanatory variables as in the connectance model. Because resource-overlap values are specific to a species at a given site, we included both site and species as random effects.

We carried out sensitivity analyses, assessing the robustness of our network metrics against changing network size by adding a network size metric (sum of nodes) to all models as a covariate and assessing the impact of missing-ness on the resource overlap metric by removing the data points that did not have an interaction with the key host recorded at a given site. The structure of the models remained otherwise the same.

We implemented all models in the brms package, which interfaces with Stan, and ran four Markov chains of 5000 iterations each, including 2000 warmup iterations. In all models we used weakly informative priors (normal(0,5) and normal(0,2) for lognormal and Bernoulli distributions, respectively) for the parameters of explanatory variables, default priors for the intercept (student(3, 0.3, 2.5)) and an exponential(1) prior for the standard deviation of the random intercept. We calculated the probability of direction (pd) for each explanatory variable using the ‘bayestestR’ package (Makowski *et al.* 2019). We consider estimates with pd equal or greater than 0.95 as likely and greater than 0.97 as very likely (Makowski *et al.* 2019).

# References:

Beyer, N., Gabriel, D. & Westphal, C. (2021). Contrasting effects of past and present mass-flowering crop cultivation on bee pollinators shaping yield components in oilseed rape. *Agric. Ecosyst. Environ.*, 319, 107537.

Blüthgen, N. & Staab, M. (2024). A Critical Evaluation of Network Approaches for Studying Species Interactions. *Annu. Rev. Ecol. Evol. Syst.*, 55, 65–88.

Bürkner, P.C. (2017). brms: An R package for Bayesian multilevel models using Stan. *J. Stat. Softw.*, 80.

Dormann, C.F., Frund, J., Bluthgen, N. & Gruber, B. (2009). Indices, Graphs and Null Models: Analyzing Bipartite Ecological Networks. *Open Ecol. J.*, 2, 7–24.

Fenton, A., Streicker, D.G., Petchey, O.L. & Pedersen, A.B. (2015). Are all hosts created equal? Partitioning host species contributions to parasite persistence in multihost communities. *Am. Nat.*, 186, 610–622.

Figueroa, L.L., Grab, H., Ng, W.H., Myers, C.R., Graystock, P., McFrederick, Q.S., *et al.* (2020). Landscape simplification shapes pathogen prevalence in plant-pollinator networks. *Ecol. Lett.*, 23, 1212–1222.

Fründ, J., Mccann, K.S. & Williams, N.M. (2016). Sampling bias is a challenge for quantifying specialization and network structure: Lessons from a quantitative niche model. *Oikos*, 125, 502–513.

Jordano, P. (1987). Patterns of Mutualistic Interactions in Pollination and Seed Dispersal: Connectance, Dependence Asymmetries, and Coevolution. *Am. Nat.*, 129, 657–677.

Makowski, D., Ben-Shachar, M. & Lüdecke, D. (2019). bayestestR: Describing Effects and their Uncertainty, Existence and Significance within the Bayesian Framework. *J. Open Source Softw.*, 4, 1541.

Manley, R., Doublet, V., Wright, O.N., Doyle, T., Refoy, I., Hedges, S., *et al.* (2023). Conservation measures or hotspots of disease transmission? Agri-environment schemes can reduce disease prevalence in pollinator communities. *Philos. Trans. R. Soc. B Biol. Sci.*, 378, 20220004.

Maurer, C., Schauer, A., Yañez, O., Neumann, P., Gajda, A., Paxton, R.J., *et al.* (2024). Species traits, landscape quality and floral resource overlap with honeybees determine virus transmission in plant–pollinator networks. *Nat. Ecol. Evol.*, 8, 2239–2251.

Patefield, W.M. (1981). Algorithm AS 159: An Efficient Method of Generating Random R × C Tables with Given Row and Column Totals. *Appl. Stat.*, 30, 91.

Proesmans, W., Albrecht, M., Gajda, A., Neumann, P., Paxton, R.J., Pioz, M., *et al.* (2021). Pathways for Novel Epidemiology: Plant–Pollinator–Pathogen Networks and Global Change. *Trends Ecol. Evol.*, 36, 623–636.

Song, C., Rohr, R.P. & Saavedra, S. (2017). Why are some plant–pollinator networks more nested than others? *J. Anim. Ecol.*, 86, 1417–1424.

Stewart Merrill, T.E., Cáceres, C.E., Gray, S., Laird, V.R., Schnitzler, Z.T. & Buck, J.C. (2022). Timescale reverses the relationship between host density and infection risk. *Proc. R. Soc. B*, 289.
